# Fiber Reinforcement Effects on Coal Gangue Concrete: A Review of Mechanical Properties, Durability and Sustainability

**DOI:** 10.3390/ma19102120

**Published:** 2026-05-18

**Authors:** Xia Qin, Sakdirat Kaewunruen, Xiangsheng Liu, Junwu Xia

**Affiliations:** 1 State Key Laboratory for Geomechanics and Deep Underground Engineering, China University of Mining and Technology, Xuzhou 221116, China; tbh683@cumt.edu.cn; 2 Birmingham Centre for Railway Research and Education, University of Birmingham, Edgbaston B15 2TT, UK; s.kaewunruen@bham.ac.uk; 3 School of Chemical and Biomolecular Engineering, The University of Sydney, Darlington 2008, Australia; xiangsheng.liu@sydney.edu.au

**Keywords:** CG, fiber-reinforced concrete, sustainability, durability, mechanical properties

## Abstract

**Highlights:**

- Reviews 82 studies on fiber-reinforced coal gangue concrete published in 2009–2026.
- Steel, polypropylene, and basalt fibers improve strength and durability differently.
- Recommended ranges are 35–70% coal gangue replacement with optimized fiber dosages.
- Crack bridging and ITZ densification explain most observed performance gains.
- CEI and CCI allow integrated assessment of strength, cost, and carbon.

**Abstract:**

Coal gangue, a by-product of coal mining, has attracted increasing attention as an alternative aggregate in concrete because it can reduce natural aggregate consumption and promote solid-waste utilization. However, its porous, heterogeneous, and source-dependent nature often leads to weak interfacial bonding, reduced strength, and poor durability. Fiber reinforcement has been widely investigated as an effective strategy to compensate for these deficiencies, but the reported results remain scattered because of differences in gangue source, replacement level, fiber type and dosage, and test methods. This review systematically synthesizes the effects of fiber reinforcement on the mechanical performance, durability, and sustainability of coal gangue concrete. Reported results are comparatively analyzed as functions of coal gangue replacement level and fiber dosage. Two strength-normalized sustainability indicators, namely the Cost Coefficient Index (CCI) and Carbon Emission Index (CEI), are further introduced to compare the economic and environmental efficiency of different mixtures. Relatively favorable overall performance was most frequently reported within the commonly reported coal gangue replacement range for coarse aggregates, about 35–70%, with steel fiber and basalt fiber commonly showing favorable intervals of about 0.8–1.0% and 0.12–0.15%, respectively; polypropylene fiber also appeared favorable at about 0.6–1.0%, although the supporting evidence is comparatively less extensive.

## 1. Introduction

As urbanization and infrastructure development continue to accelerate, the construction industry faces increasing pressure to secure material supply while reducing environmental burdens [1]. Among the various industrial solid wastes generated by mining activities, coal gangue (CG), a by-product of coal extraction, has attracted considerable attention because of its large stockpiles and underutilized resource potential [2]. Improper disposal of CG not only occupies land and threatens environmental safety, but may also cause long-term ecological and public health risks [3]. In response, growing efforts have been devoted to the reuse of CG in construction materials [4,5]. Recent studies have shown that CG can be used as a partial or full replacement for natural aggregates in concrete as coal gangue coarse aggregates (CGA) or coal gangue fine aggregates (CGS) [6,7]. Compared with the traditional view of CG as a low-grade substitute, properly processed and proportioned coal gangue concrete (CGC) can satisfy the basic mechanical and durability requirements of many civil engineering applications [8,9]. In addition, because CG is abundant and inexpensive, its use in concrete offers potential benefits in cost reduction, natural resource conservation, and embodied-carbon mitigation, which is consistent with the principles of the circular economy and carbon neutrality [10,11].

Nonetheless, the direct use of untreated and non-calcined CGA in concrete still involves several limitations. CGA often exhibits irregular particle shapes, high porosity, and heterogeneous chemical compositions, which may increase water demand and weaken the interfacial transition zone (ITZ) between the aggregate and cement paste [12,13]. These localized weaknesses can adversely affect the long-term performance of concrete under mechanical loading or in aggressive environments [14,15]. In this context, fiber reinforcement has emerged as a feasible and effective strategy for further improving material performance while retaining the economic and environmental advantages of CG utilization [16]. It should be noted that fiber reinforcement is not intended to compensate for an inherently unsuitable aggregate system, but rather to enhance the behavior of a basically serviceable concrete material [17]. Particularly where CG is abundantly available, its in situ use in concrete construction can offer clear logistical and economic advantages by reducing material transportation and disposal costs [18]. However, these applications often require concrete with improved crack resistance, flexural capacity, and long-term durability, especially under harsh conditions such as sulfate attack and freeze–thaw cycling [19]. Under such circumstances, the incorporation of steel fibers (SF), polypropylene fibers (PPF), and basalt fibers (BF), which are the main fiber types discussed in the currently available FRCGC literature, provides a practical route to improve the performance limitations of CGC while retaining its sustainability-related advantages.

Fiber-reinforced concrete (FRC) has demonstrated clear advantages in crack-bridging ability, impact resistance, ductility, and durability [20,21]. In CGC, fiber reinforcement is of particular significance because CGA are often associated with low strength, high porosity, and limited durability [22]. Accordingly, the development of fiber-reinforced CG concrete (FRCGC) offers a promising route to combine the resource-utilization benefits of CG with the mechanical advantages of fiber technology. Nevertheless, because CG is inherently a low-cost and low-carbon industrial solid waste [23], the effectiveness of fiber incorporation should be evaluated not only by performance enhancement but also by its economic and environmental implications. Although previous studies have confirmed the strengthening and toughening effects of fibers in concrete, the trade-offs between mechanical improvement and sustainability performance in FRCGC remain insufficiently synthesized.

## 2. Research Significance and Methodology

### 2.1. Significance

**Figure 1:**
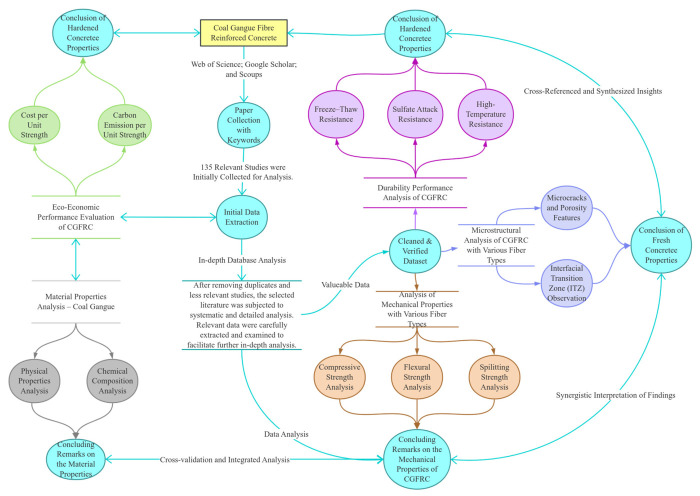
Flowchart of the literature search and review process.

FRCGC provides a realistic pathway for the large-scale utilization of CG in concrete, with potential benefits in resource conservation, cost reduction, and embodied-carbon mitigation [24,25]. However, the existing evidence remains difficult to translate into practical guidance because current studies are highly scattered in terms of gangue source, replacement level, fiber type and dosage, and testing methodology. In addition, most available research has focused primarily on performance enhancement, whereas the economic and environmental implications of fiber incorporation have rarely been discussed in a comparable manner [26]. Therefore, the practical suitability of FRCGC for sustainable engineering applications remains insufficiently clarified. This review addresses these issues by providing a systematic synthesis of the effects of fiber reinforcement on CG concrete, with particular emphasis on mechanical performance, durability, and sustainability. Reported results are comparatively discussed according to CG replacement level and fiber dosage to identify recurring trends and representative reinforcement ranges. In addition, strength-normalized indicators related to cost and embodied carbon are introduced to further compare the economic and environmental efficiency of different mixtures. The overall search strategy, data flow, and thematic framework adopted in this study are presented in Figure 1.

### 2.2. Knowledge Map and Research Timeline of FRCGC

To provide a supplementary overview of the research landscape of FRCGC, a brief bibliometric analysis was conducted using CiteSpace. This analysis was intended to identify major research themes, track their temporal evolution, and support the overall understanding of research trends in this field. It should be noted that the bibliometric results serve only as a complementary overview, whereas the main body of this review is based on a critical synthesis of published findings on mechanical performance, durability, and sustainability.

The dataset was retrieved from the Web of Science Core Collection using the search terms “Coal gangue,” “fiber,” and “concrete,” covering the period from 2009 to 2026. A total of 82 publications were collected and analyzed. After the initial retrieval, the literature was screened in a stepwise manner to ensure both thematic relevance and analytical usability. Duplicate records and evidently unrelated publications were excluded. The remaining studies were further examined with reference to three main criteria: direct relevance to FRCGC, completeness of the reported experimental data, and clarity of the material characterization and testing methodology. Only publications providing sufficiently identifiable information on mixture design, test variables, and performance results were retained for comparative synthesis. No formal filtering was performed on the basis of whether the reported conclusions were favorable or unfavorable; instead, the selection focused on the relevance of the study and the availability of interpretable experimental information. Ultimately, 82 publications were included in the final review.

**Figure 2:**
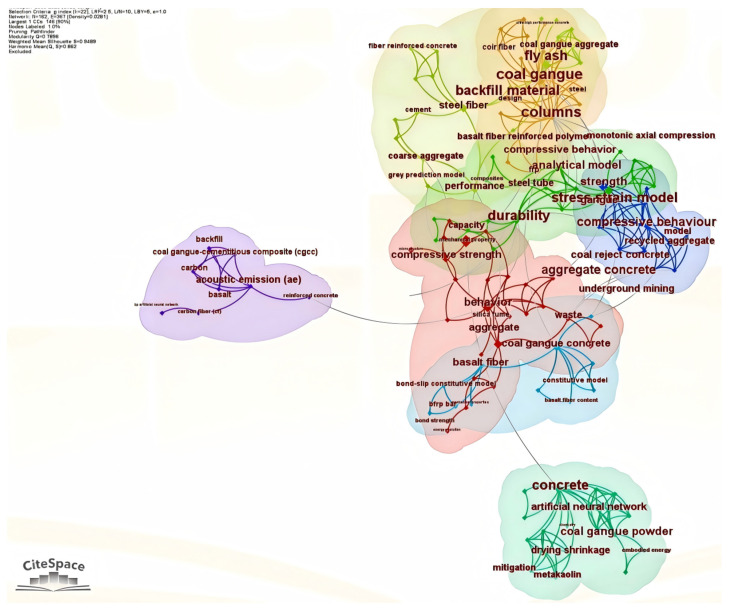
Keyword co-occurrence map of FRCGC-related research.

**Figure 3:**
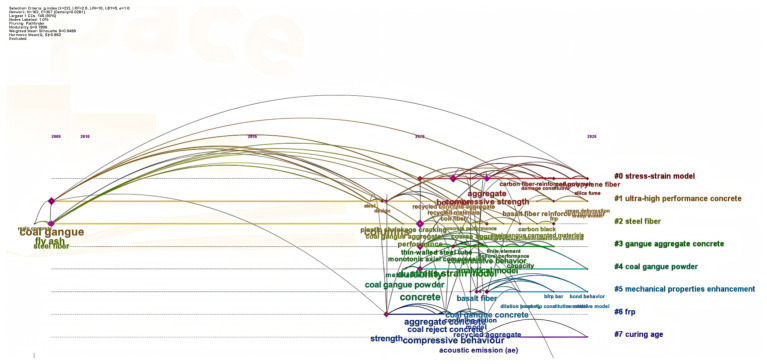
Clustered timeline view of FRCGC-related research.

After the screening process was completed, the final dataset was used for the subsequent bibliometric and comparative analysis. Two visual outputs were generated: (1) a keyword co-occurrence map (Figure 2), showing the major conceptual focuses of the field, and (2) a clustered timeline view (Figure 3), illustrating the evolution of key research themes over time. The bibliometric analysis was intended to provide a supplementary overview of the retrieved literature and to identify the principal research themes in this field. Within this dataset, the mapped results suggest that FRCGC research has mainly focused on CG utilization, fiber modification, strength development, and durability performance, which also guide the thematic organization of the following sections.

The keyword co-occurrence map highlights several closely connected themes in the retrieved FRCGC literature, indicating that research in this area has gradually expanded in recent years. The most prominent keywords are associated with CG utilization, fiber reinforcement, compressive strength, durability, and concrete performance, suggesting that current studies are mainly concerned with improving the performance of CG concrete while enhancing its utilization potential through fiber modification. The frequent co-occurrence of terms related to CGA, SF, BF, and strength development further shows that FRCGC has gradually emerged as a research topic linking solid waste utilization with concrete performance enhancement. At the same time, the keyword structure suggests that the focus of this field is no longer limited to strength improvement alone. In addition to conventional mechanical indicators, increasing attention has also been given to durability, fiber–matrix interaction, and microstructural behavior, indicating a gradual shift from isolated mechanical evaluation toward a more integrated understanding of performance, mechanism, and long-term applicability.

Figure 3 further emphasizes the temporal evolution of FRCGC-related research within the retrieved dataset from 2009 to 2026. Unlike the keyword co-occurrence map, which highlights thematic connections, the timeline clustering view shows how the focus of this field has shifted over time. Early studies were mainly centered on stress–strain behavior, compressive response, and constitutive analysis, whereas later research has gradually expanded toward fiber modification, durability improvement, microstructural interpretation, and sustainability evaluation. This temporal pattern suggests that the development of FRCGC research has moved from basic mechanical characterization toward a broader and more application-oriented understanding of performance, mechanism, and long-term serviceability. SF remains the most extensively studied reinforcement, followed by BF and PPF. These findings are also consistent with the thematic focus of the present review, which examines the reinforcement effects of different fibers on the mechanical performance, durability, and sustainability of FRCGC.

### 2.3. Review Scope, Aims, and Analytical Objectives

After clarifying the research significance and methodology, this section defines the scope and analytical objectives of the present review. This review focuses on FRCGC, with particular attention to the material characteristics of CG reported in concrete-related studies, the reinforcement effects of different fiber types, the relationships between macroscopic performance and microstructural mechanisms, durability behavior under representative aggressive conditions, and the sustainability-related efficiency of different mix configurations. The main objectives of this review are as follows:

To summarize the key physical and chemical properties of CG that influence its use in concrete and to clarify their implications for the performance of FRCGC;

To compare the reinforcement effects of different fiber types and dosages on the mechanical behavior of CG concrete, with emphasis on compressive, splitting tensile, and flexural performance;

To relate the observed macroscopic behaviors to microstructural features, including ITZ quality, fiber dispersion, crack development, and pore structure evolution;

To evaluate the durability performance of FRCGC under representative aggressive conditions, including freeze–thaw cycling, sulfate attack, elevated temperature exposure, and chloride ingress;

To assess the economic and environmental efficiency of different FRCGC mixtures using strength-normalized indicators of cost and embodied carbon.

## 3. Review of CG Properties

**Figure 4:**
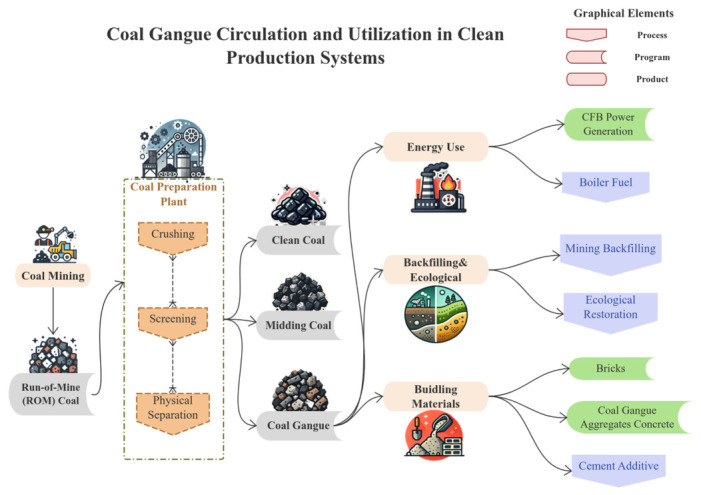
Cleaner production framework for CG waste utilization pathways.

CG is a solid waste generated during coal mining and washing. Its long-term accumulation has caused serious environmental problems, including land occupation, spontaneous combustion, leachate pollution, and geological instability [27,28]. With increasing emphasis on resource conservation and environmentally responsible development, the utilization of CG has become an important component of sustainable transformation in the coal industry [29]. For a better understanding of the main utilization pathways of CG, Figure 4 presents a synthesized schematic based on the reviewed literature. As illustrated, CG can be reused in several fields after crushing, screening, and separation, including energy recovery, ecological restoration, and construction applications [14,30]. Among these utilization pathways, the use of CG in concrete has attracted increasing attention in recent years, particularly in fiber-reinforced systems [8]. Owing to its silica- and alumina-containing mineral composition, CG can be used as a supplementary cementitious component or as a substitute for natural aggregates under appropriate conditions [22,31]. Nevertheless, inherent drawbacks such as high porosity, low mechanical strength, and unstable reactivity limit its stand-alone application in structural concrete. In this context, the synergistic enhancement of CG concrete using various fibers has emerged as a promising approach to improve its overall performance, as previously outlined.

At the same time, the physical and chemical properties of CG used in concrete vary significantly depending on geological origin, mineral composition, and material condition [32]. In the present review, the discussion mainly concerns CG reported in concrete-related studies, since this allows a more consistent basis for comparing the effects of fiber reinforcement. Other gangue types or application pathways with more complex treatment backgrounds are not discussed in detail. Such variability directly affects its workability, mechanical behavior, durability, and overall suitability for concrete applications. Therefore, a clear understanding of the key properties of CG is essential for its rational use in FRCGC.

### 3.1. CG: Physical Properties and Engineering Implications

Significant regional differences in CG’s physical properties have been observed, primarily due to variations in depositional environments, mineral compositions, and chemical constituents. To provide a broader understanding of its engineering properties, this study compiles data reported by Chinese researchers and researchers from other countries into two datasets presented in Table 1 and Table 2. Although the specific indicators reported in the literature are not completely uniform, both tables describe the fundamental characteristics of CG that are relevant to its engineering behavior. Therefore, the purpose of this comparison is not to establish a strict point-by-point correspondence between individual parameters, but rather to outline the overall property range, material tendencies, and potential implications for the use of CG in construction materials, especially concrete-related applications. This approach helps provide a more comprehensive reference for evaluating the rational utilization of CG in engineering materials.

Analysis of data presented in Table 1 and Table 2 reveals that the average bulk density of CG from China is 2477 kg/m^3^, which is close to that of limestone (2600–2700 kg/m^3^), though still generally lower than natural aggregates [51]. This suggests a relatively low particle mass and the possible presence of internal porosity. In contrast, the average specific gravity of international CG samples is 2.07, significantly lower than that of conventional stone materials (2.5–2.7), with considerable variation across countries. For instance, CG from China exhibits the highest specific gravity (2.54), closely resembling natural aggregates, whereas Indian samples show the lowest value (1.81), indicating high porosity and low compactness—characteristics that may limit their suitability for high-strength structural applications. From an engineering perspective, CG with higher density, such as that from China, is more appropriate for use as coarse aggregate in concrete, while lower-density gangue may be considered for use in lightweight fillers or insulation materials, subject to appropriate mechanical enhancement.

Water sensitivity is another critical factor influencing the engineering applicability of CG, particularly affecting mixing water demand, material durability, and the stability of road base applications. According to Table 1, the average water absorption rate of CG from China is 3.97%, significantly higher than that of typical natural stone (0.5–2%) [51], with some samples exceeding 11%, indicating pronounced internal porosity. Table 2 provides further evidence of source-dependent water sensitivity through indicators such as liquid limit, with an average value of 31.78%. Indian CG exhibits the highest liquid limit (35.5%), suggesting strong water absorption potential and risk of swelling or softening under wet conditions, whereas Australia samples show the lowest (27.2%), indicating relatively stable water behaviors. Although the liquid limit is not directly equivalent to water absorption in concrete aggregate evaluation, it still reflects the tendency of CG to respond to moisture and therefore helps explain differences in wet-state stability among sources [3]. Taken together, these results suggest that the water sensitivity of CG is strongly source dependent and should be carefully considered when evaluating its suitability for construction materials.

Mechanical performance is a key determinant of CG’s applicability in structural materials and is commonly assessed using the crushing value or plasticity index. As shown in Table 1, the average crushing value of the reported CG from China is 16.39%, which falls within a generally acceptable range for aggregate use and suggests moderate mechanical stability, although some samples exhibit higher values (up to 22.6%), indicating that additional treatment or strengthening may be required before use in structural concrete. Table 2 provides supplementary information on source-dependent material stability through indicators such as plasticity index. The average plasticity index of the international datasets is 10.26, with Australia CG showing relatively lower values. Although the plasticity index is not directly equivalent to crushing value in the evaluation of concrete aggregates, it still helps reflect the deformation sensitivity and stability characteristics of CG under engineering use. Taken together, these results suggest that the mechanical applicability of CG is strongly source dependent, and that its suitability for concrete should preferably be assessed using aggregate-related parameters directly relevant to concrete performance.

Moreover, permeability is a critical parameter influencing the suitability of CG for impermeable liners, drainage layers, and foundation stabilization. Although direct permeability data are not available in the China datasets summarized in Table 1, the reported water absorption values range from 1.10% to 11.40%, with an average of 3.97%. Some reported values, such as 11.4% [34] and 7.14–7.38% [41], are markedly higher than the typical range reported for natural stone (0.5–2%) [51], which may suggest a relatively more open pore structure and greater sensitivity to water ingress. Table 2 shows that the reported permeability of international CG varies from 10^−8^ to 10^−5^ m/s, again indicating strong source dependence. While permeability is more commonly discussed in geotechnical contexts, these data still help explain the moisture-transport tendency of CG and its possible implications for durability-related behavior in engineering materials.

In summary, the physical properties of CG are strongly source dependent, and regional variability leads to clear differences in suitability for fiber-reinforced concrete applications. CG reported in many studies from China generally exhibits higher density, moderate water absorption, lower crushing value, and relatively low permeability. These characteristics indicate better potential as coarse aggregate in structural concrete, where load-bearing capacity, stiffness, and durability retention are required. By contrast, CG reported in Indian studies is often less dense and more porous, suggesting greater suitability for lightweight concrete, nonstructural components, or thermal insulating applications, unless additional processing is adopted. When CG shows high water absorption, the mix design should explicitly address water demand through moisture conditioning and careful control of the water-to-binder ratio, and may benefit from supplementary cementitious materials or surface treatments to limit porosity and improve interfacial quality. Variations in permeability and plasticity further imply that CG can be selected or processed for targeted engineering needs, such as improving impermeability or maintaining flowability in self-compacting and highly workable concrete systems.

### 3.2. The Chemical Composition and Reactivity Potential of CG

The chemical composition of CG substantially determines its potential application in the construction materials sector. In particular, when used as a cementitious additive or reactive filler, essential properties such as pozzolanic activity, binding capacity, and durability are directly influenced by its chemical makeup. Therefore, a systematic evaluation of CG chemistry across different regions is crucial for assessing its engineering suitability and optimizing resource utilization strategies in a global context.

However, the chemical composition of CG is shaped by a complex interplay of geological factors, including parent rock type, sedimentary environment, and diagenetic history, giving rise to significant regional variability. In addition, variation in analytical methods and reporting standards across countries may further complicate cross-regional data comparison.

To address these challenges and provide a comprehensive understanding of its chemical characteristics, representative datasets from China, Australia, India, Nigeria, Pakistan, Poland, Spain, and the United Kingdom were compiled and standardized into a unified format, as presented in Table 3. Table 3 includes major oxide components such as SiO_2_, Al_2_O_3_, Fe_2_O_3_, CaO, MgO, K_2_O, Na_2_O, and Loss on Ignition (LOI), offering a consistent framework for analyzing global distribution patterns and evaluating the material’s potential for cementitious and pozzolanic applications.

The pozzolanic activity and cementitious potential of CG are primarily governed by the combined presence of SiO_2_, Al_2_O_3_, and CaO. As shown in Table 3, SiO_2_ remains the dominant component, with an average content of 54.21%, while Al_2_O_3_ averages 21.1%, forming the core of the reactive silico-aluminate matrix. High SiO_2_ and Al_2_O_3_ content—particularly in samples from China and Nigeria—suggests strong pozzolanic reactivity, favorable for use as supplementary cementitious materials. Notably, the gangue samples from China also exhibit relatively high CaO levels (up to 7.58%), providing additional latent hydraulic activity. In contrast, the Pakistani samples contain the lowest SiO_2_ (31.37%) and CaO (0.08%) values, indicating limited intrinsic reactivity.

In addition to reactivity, the long-term performance and stability of CG are influenced by secondary oxides such as Fe_2_O_3_ and loss on ignition (LOI). Samples from China exhibit the highest Fe_2_O_3_ content (8.02%), which may enhance resistance to sulfate attack and contribute to darker coloration in concrete. Conversely, Nigerian gangue shows the lowest Fe_2_O_3_ level (3.13%), making it more suitable for lightweight, non-structural applications. LOI, which reflects the content of volatile matter, organics, and carbonates, varies significantly among samples. Pakistani gangue demonstrates an exceptionally high LOI (41.84%), indicating poor thermal stability and potential negative effects on concrete durability. In contrast, samples from China with moderate LOI (10.79%) are better suited to standard cementitious applications. The wide variation in Fe_2_O_3_ and LOI between samples indicates disparities in thermal stability and potential durability, which may influence their suitability in specific environmental conditions.

Based on the combined analysis of major and minor oxides, CG can be broadly classified into several application-oriented categories. Samples with high SiO_2_ and Al_2_O_3_ content and moderate CaO levels (e.g., those from China and Nigeria) are most suitable for use as pozzolanic or blended cementitious materials. Materials with high LOI and low reactivity—such as those from Pakistan—are less suitable for cementitious applications but may have value in lightweight fills, geotechnical amendments, or thermal insulation layers.

Overall, the chemical composition of CG shows clear source-dependent patterns, which directly affect its engineering potential in cementitious systems. CG with relatively high SiO_2_ and Al_2_O_3_ contents, as reported for samples from China and Nigeria, is more promising for pozzolanic utilization after appropriate activation, contributing to matrix densification and improved interfacial quality. Gangue with higher CaO content, observed in some samples from China, may provide additional hydraulic reactivity and thus greater binder potential, which can benefit early strength development. The Fe_2_O_3_ content may influence durability-related reactions and performance in aggressive environments, particularly where sulfate exposure is relevant. The LOI should be carefully assessed, especially for samples reported from Pakistan, because high LOI may indicate unburned carbon or volatile components that can impair workability, admixture efficiency, and long-term performance. In such cases, thermal or chemical pretreatment may be required before CG is used in structural concrete.

### 3.3. Concluding Remarks

The physical and chemical properties of CG are strongly source dependent, and regional variability leads to clear differences in engineering applicability and structural suitability. In many reported cases, CG with higher density, lower water absorption, and better mechanical stability shows greater potential as coarse aggregate for structural concrete, whereas more porous and lower density gangue is more aligned with lightweight or nonstructural uses unless additional processing is applied. Chemically, CG is typically dominated by SiO_2_ and Al_2_O_3_, while variations in CaO, Fe_2_O_3,_ and LOI influence reactivity, interfacial quality, and durability-related risks. These observations indicate that reliable use of CG in structural concrete requires region-specific characterization and consideration of combined physical and chemical effects, rather than reliance on a single parameter. For clarity and comparability, Table 4 summarizes the key material property trends reported in the literature and highlights the main gaps.

## 4. Comparative Analysis of Different Fiber Types in CGC

**Figure 5:**
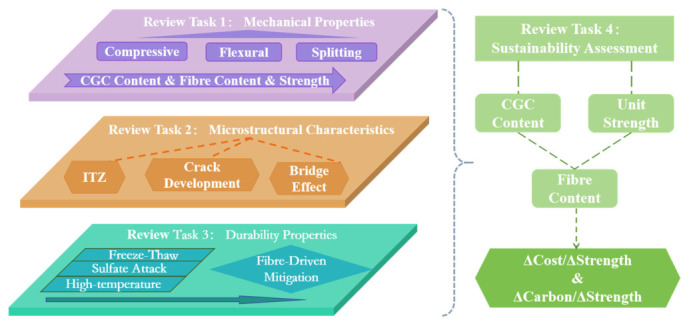
Comparative Framework for FRCGC.

To systematically evaluate the role of fibers in enhancing the performance of FRCGC, this review adopts a classification-based approach in which each fiber type is examined independently, as shown in Figure 5. The analysis is structured around two main dimensions: (i) mechanical performance—including compressive, tensile, and flexural strength; and (ii) microstructural characteristics—such as interfacial bonding, crack propagation paths, and internal morphology observed via Scanning Electron Microscopy(SEM). Among the various fiber types explored in the current literature, three categories—BF, SF, and PPF—have been most thoroughly studied and so form the primary focus of this section. By analyzing their respective roles in improving FRCGC properties, this review aims to provide a comparative understanding of the interaction between CG-based matrices and different fiber materials. Furthermore, performance differences across fiber types will be interpreted with reference to the aforementioned physical and chemical properties of CG, offering material-based explanations for observed behaviors and property variations.

### 4.1. Properties of CGC

Before comparing the reinforcing effects of different fibers, the baseline characteristics of coal gangue concrete (CGC) should first be clarified in light of the intrinsic properties of CG discussed in Section 3. As shown by the compiled datasets in this study, untreated CG generally exhibits relatively high water absorption, considerable crushing susceptibility, porous internal structure, and marked source-dependent variability, all of which directly affect the matrix behavior of non-fiber CGC.

This understanding is consistent with the review of Gao et al. [8] who pointed out that CG usually has an apparent density close to natural stone, but a lower bulk density, together with relatively high water absorption and crushing value. Accordingly, when used as coarse aggregate, it is mechanically more fragile and more likely to induce failure within the gangue particles themselves or in the adjacent ITZ. Gao et al. [8] further summarized that increasing gangue replacement generally reduces the workability, compressive strength, elastic modulus, and splitting tensile strength of concrete, while the adverse influence on durability is often even more pronounced because the porous structure of gangue provides additional transport pathways for air and liquid. Their review also showed that, under full coarse aggregate replacement, the compressive strength of CGC commonly decreased by about 15–20%, and the elastic modulus generally showed a declining trend with increasing gangue replacement.

A more recent synthesis by Wang et al. [58] further extends this interpretation by emphasizing that the key non-fiber drawback of CG lies in the combined effect of high porosity, high water absorption, and low aggregate strength. Their review reported that the porosity of CGA can be 3–5 times that of natural aggregate, its water absorption is typically 5.0–9.0%, and its crushing value commonly falls within 16.0–23.0%. More importantly, they proposed a progressive degradation mechanism from raw material defect to concrete response, indicating that the high porosity of CG promotes water absorption and non-uniform moisture redistribution, thereby weakening the ITZ and ultimately resulting in reductions in overall strength and durability. Wang et al. [58] also highlighted the strong source dependence of CG chemistry. Although the compositional ranges reported in their review are not identical to those compiled in the present study, both datasets consistently indicate substantial variability in SiO_2_ and Al_2_O_3_, which helps explain why the performance of CGC cannot be generalized without proper source characterization.

From the mechanical perspective, the available CGC evidence suggests that the influence of CG on CGC is not limited to simple strength loss, but also involves stiffness reduction and higher variability in failure mode [59]. As summarized in the Section 3 dataset, the physical properties of untreated CG span a relatively wide range, with water absorption in the China-based dataset averaging 3.97% and some samples exceeding 11%, while crushing values are commonly around 16–19% but may reach 22.6%. Correspondingly, in non-fiber CGC systems, higher gangue replacement is generally associated with reduced workability, lower compressive strength, lower elastic modulus, and weaker tensile-related performance, especially when the gangue particles are more porous or mechanically weaker. In this sense, the role of fiber reinforcement in the following sections should not be interpreted in isolation; rather, fibers should be understood as acting on a matrix that has already been altered by the intrinsic weaknesses of gangue. Their main function is therefore to compensate for or mitigate the defects already introduced by CG in non-fiber concrete, rather than to replace the role of matrix design itself.

### 4.2. Basalt Fiber Reinforcement in CGC

In recent years, basalt fiber-reinforced concrete (BFRC) has attracted increasing attention due to its excellent mechanical properties and durability, making it widely applicable for use in pavements, structural components, and seismic retrofitting [60,61]. Numerous studies have demonstrated that the inclusion of BF enhances tensile and flexural strength while significantly improving crack resistance and impact tolerance [62,63]. In contrast, CG—a major solid waste from the coal industry—often exhibits poor aggregate strength and high porosity, leading to reduced mechanical performance and durability when incorporated within concrete. To address these limitations, a growing body of research has explored the synergistic use of BF and CGA, aiming to leverage fiber reinforcement to compensate for the structural deficiencies of gangue-based concrete. This section reviews key experimental studies, summarizing their findings on fiber dosage, gangue replacement ratios for coarse aggregates, and mechanical outcomes, and provides a comparative analysis to inform the development of sustainable, high-performance concrete systems incorporating CG.

#### 4.2.1. Mechanical Properties of BFRCGC

To facilitate cross-study comparison of test outcomes related to fiber content, CG replacement level (*R_cg_*), and mechanical performance, this review compiles a series of representative studies of basalt fiber reinforced CG concrete (BFRCGC) with key experimental parameters and results summarized in Table 5.

Overall, most studies converge on an optimal fiber content range of approximately 0.1% to 0.2%. For example, Zhu et al. [22] report that compressive strength increased to 34.52 MPa at 0.15% fiber content but declined noticeably at 0.18%. Similarly, He et al.’s [39] experiments, which adopted a target strength of approximately 32 MPa, found that compressive strength showed slight improvement at low-to-moderate dosages, while splitting and flexural strength improved more significantly. However, further increases in fiber content led to reduced workability and mixing difficulties due to fiber agglomeration. Notably, Yang and Zha [64] achieved a peak compressive strength of up to 50.02 MPa when concrete fiber content exceeded 0.25%, but this was not accompanied by proportional gains in splitting or flexural strength—likely due to poor dispersion and weak localized bonding. These findings indicate that excessive fiber content or fiber length often negates the overall mechanical benefits of fiber reinforcement when the CG replacement ratio for coarse aggregates reaches 40%.

Across the reviewed studies, the compressive strength of BFRCGC systems generally ranged between 20 MPa and 40 MPa, reflecting variations in binder composition, aggregate quality, and mix design strategies. Despite these differences, a common trend can be identified: when the *R_cg_* was maintained at around 40% and the BF content was within a range of 0.1% to 0.15%, compressive strength typically increased by 5% to 15%. Zhu et al. [22] provide a representative example of this trend, where compressive strength rose from 30.68 MPa at 0.1% fiber content to 34.52 MPa at 0.15%. However, a further increase to 0.18% resulted in a drop to 31.18 MPa, highlighting the diminishing returns and even adverse effects of excessive fiber reinforcement. This non-linear response suggests that the presence of excessive fibers may lead to agglomeration and increased porosity, ultimately offsetting the reinforcement benefits initially achieved at optimal dosages. A similar trend was observed in He et al. [39], where compressive strength plateaued or declined once fiber content exceeded 0.2%, mainly due to poor dispersion. Al-Kharabsheh et al. [65] further noted that when fiber content exceeded 0.3%, fiber entanglement and paste absorption intensified, impeding matrix densification and complicating casting and compaction.

Among the three mechanical indicators studied, flexural strength appears to be the most sensitive to fiber reinforcement. As shown in He et al. [39] and Yang and Zha [64], when fiber content ranged between 0.1% and 0.15%, flexural strength increased by 10–20%. For instance, He, et al. [39] reported that the flexural strength of the control group was 4.01 MPa, which increased to 4.53 MPa with 0.15% fiber content and 18 mm length—a gain of over 12%. Zheng et al. [66] have attributed this to the ability of BF to arrest the development of microcracks around CGA, thereby delaying crack propagation and improving flexural capacity. However, flexural strength does not increase linearly with fiber dosage. In Yang and Zha [64], although compressive strength continued to rise under high fiber content (≥0.25%), no consistent improvement in flexural performance was apparent. This may be due to two competing mechanisms: while fiber bridging helps reinforce crack-prone regions, excessive fiber content may cause agglomeration and macro-voids that compromise performance.

Splitting tensile strength provides a more direct assessment of fiber efficacy in resisting tensile stress and mitigating brittle failure. In Zhu et al.’s [22] experiments, increasing fiber content from 0.1% to 0.15% raised splitting strength from 2.09 MPa to 2.41 MPa. He et al. [39] found that the optimal combination of 0.15% content and 18 mm length resulted in a peak splitting strength of 2.80 MPa—approximately 16% higher than that of the control group. Even in Li et al. [40], where the target strength was lower, splitting strength increased from 1.81 MPa to 2.95 MPa with moderate fiber addition. These improvements are attributed to the high tensile strength and toughness of BF. Under tensile stress, microcracks in the CG matrix tend to propagate; BF anchors into the crack flanks and absorbs tensile loads, delaying fracture [67]. However, poor dispersion or excessive dosage may result in fiber clustering, leading to localized stress concentration and ineffective load transfer, thus reducing the overall reinforcing effect.

It should be noted that the mechanical differences reported for BFRCGC were not governed by BF dosage and fiber length alone, but also by the underlying mixture design. Representative studies reveal clear variations in water-to-binder ratio, cementitious composition, and the use of mineral or chemical admixtures, which partly explain why the reported strength levels are not directly comparable across studies. For example, Zhu et al. [22] employed a relatively stable cement-based system (w/c = 0.46, 424 kg/m^3^ cement, 195 kg/m^3^ water, and 2.12 kg/m^3^ water reducer), under which 0.15% BF increased the compressive strength to 34.52 MPa at 40% CGA replacement, indicating that an appropriate BF dosage can effectively improve both load-bearing capacity and crack resistance in CGC. By comparison, He et al. [39] also adopted a 40% replacement rate but incorporated silica fume and varied fiber length simultaneously; in this denser matrix system, the gain in compressive strength was relatively moderate, whereas the improvements in splitting tensile and flexural behavior were more evident, suggesting that BF contributed more strongly through crack bridging and crack-arresting mechanisms than through direct compressive strengthening. Qiu et al. [41] further showed that, at 40% replacement, the incorporation of fly ash and silica fume improved matrix compactness and ITZ quality, allowing 0.15 vol% BF to work more effectively and increasing compressive strength to 36.77 MPa. Therefore, the mechanical response of BFRCGC should be interpreted by considering both fiber parameters and mixture-related factors, rather than attributing the observed differences solely to BF dosage.

It is worth noting that the reviewed BFRCGC studies mainly employed CGA replacement ratios between 35% and 50%, with 40% being the most common level. Although this relatively narrow range improves comparability across studies, it also means that the independent role of *R_cg_* in governing mechanical behavior has not been systematically investigated. Most existing studies concentrated on the effects of BF dosage and fiber dimensions, and generally suggest that moderate BF contents of approximately 0.10%–0.15% are more beneficial, whereas excessive contents tend to reduce effectiveness because of poor dispersion and reduced workability. Nevertheless, because these observations were derived largely under similar replacement conditions, current evidence remains insufficient to clarify how variation in *R_cg_* may alter the compressive, splitting tensile, and flexural performance of BFRC systems.

#### 4.2.2. Microstructural Characteristics of BFRCGC

Microstructural analysis plays a crucial role in understanding the performance of BFRCGC, serving as a key indicator of the material’s macroscopic mechanical behaviors and durability. Numerous studies have demonstrated that the macro-level properties of FRC are closely associated with its internal microstructure—particularly the ITZ, pore distribution, and microcrack propagation [68]. The quality of the ITZ significantly influences both strength and durability, while pore structure governs permeability, frost resistance, and resistance to chemical attack [69]. Therefore, examining the microstructural mechanisms of BF in BFRCGC is essential for guiding material optimization and supporting practical engineering applications. Existing studies have shown that fiber incorporation can significantly improve the microstructure of concrete. For instance, Chen et al. [70] reported that BF creates a pronounced bridging effect within the ITZ, effectively restraining crack propagation and enhancing interface compactness. In such cases, Table 6 summarizes the key mechanisms, common findings, and variations in microstructural interpretations of BFRCGC across different studies.

As shown in Table 6, there is general agreement that moderate fiber content is crucial for maximizing structural performance, particularly in promoting crack inhibition and improving interfacial quality through the bridging effect. While specific fiber lengths and dosages vary among studies, the consensus is that excessive fiber content may cause dispersion issues and diminished efficiency. Additionally, differences in experimental frameworks—such as the use of alkali activation, blended mineral admixtures, or pervious concrete matrices—indicate the importance of material selection and condition control in BFRCGC microstructural research.

**Figure 6:**
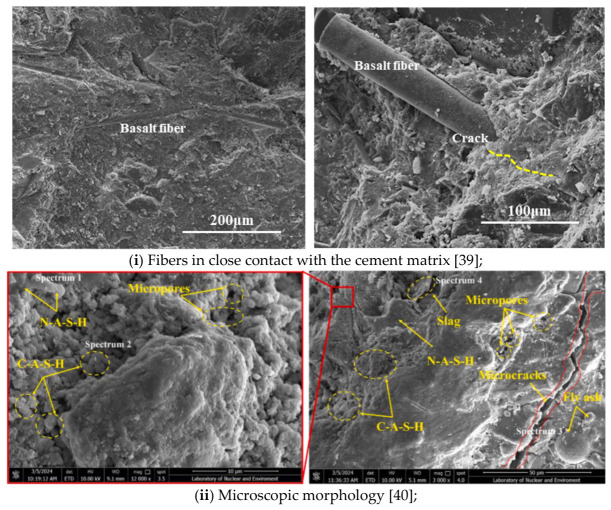

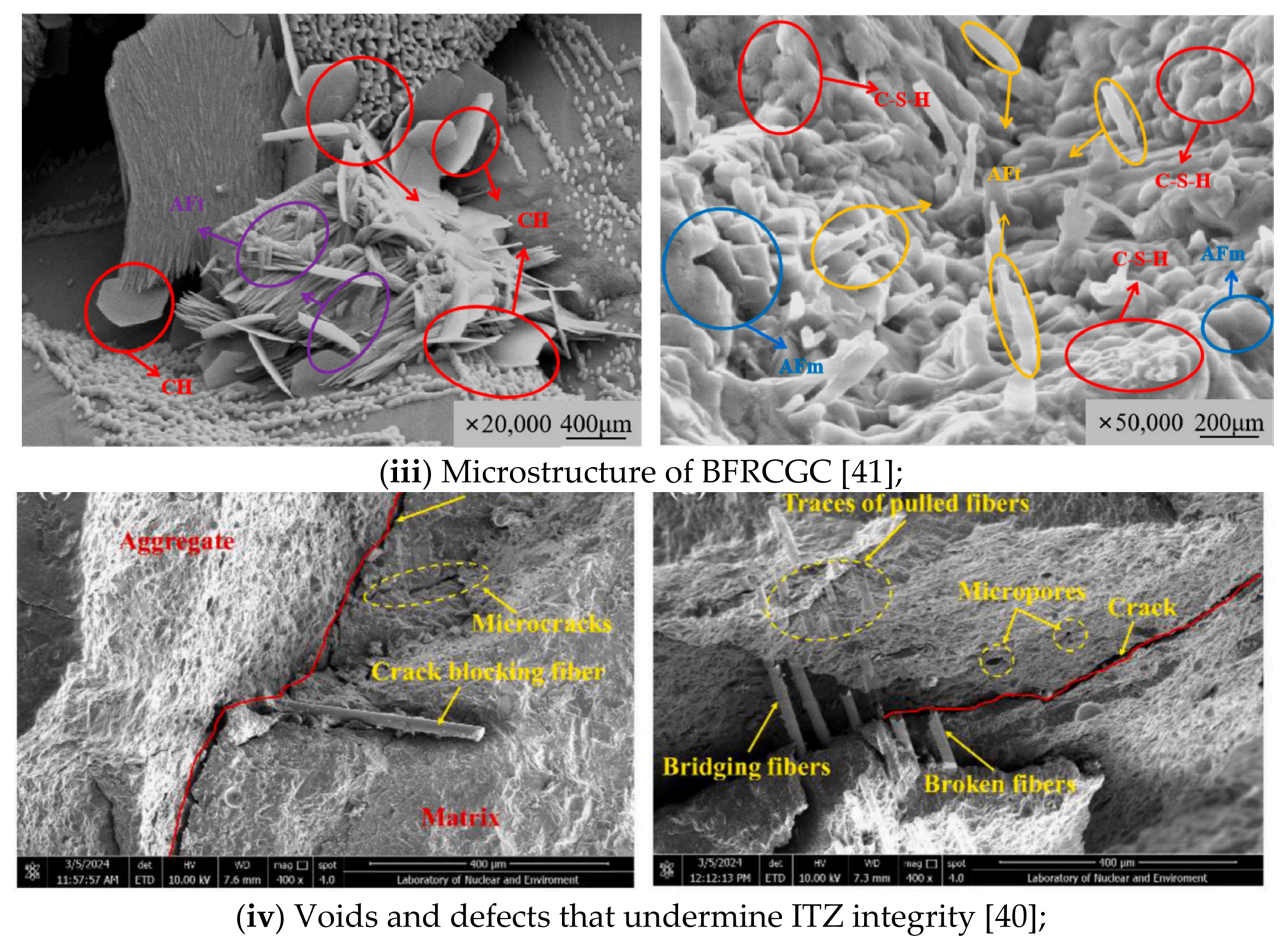
Microstructure analysis of BFRCGC.

As summarized in Table 6 and supported by SEM images (e.g., Figure 6i–iii), BF demonstrates a clear bridging effect that suppresses crack initiation and propagation, thereby increasing the compactness of the ITZ. Hydration products such as C-S-H gel and ettringite (AFt) are often observed on the fiber surface, forming a dense bonding layer that enhances fiber–matrix adhesion and strengthens interfacial performance. Moreover, Figure 6vi shows that excessive fiber content (>0.15%) or excessive fiber length (>18 mm) may lead to fiber entanglement and agglomeration, forming local voids and defects which undermine ITZ integrity. When fibers are unevenly dispersed, their bridging capacity is weakened, resulting in loose interfacial zones and reduced macroscopic performance. In contrast, optimal fiber content and dispersion facilitate the development of a continuous micro-bridging network that directly improves structural performance.

Indeed, several studies have linked these microstructural changes to macro-level strength outcomes. For instance, Zhu et al. [22] reported that a fiber content of 0.15% yielded the highest compressive strength (34.52 MPa), with both splitting and flexural strength improving with dosage—until excessive fiber addition caused agglomeration and performance decline. He et al. [39] found that while compressive strength remained stable above 32 MPa, tensile and flexural strengths significantly improved, corresponding to denser ITZs and stronger fiber–matrix bonds observed under SEM. Li et al. [40] emphasize that poor dispersion of thicker BF led to weak interfacial zones, limiting their reinforcement potential in low-strength matrices. Qiu et al. [41] confirmed that the synergy between BF and compound mineral admixtures led to pore refinement and simultaneous enhancement of mechanical and durability properties.

More specifically, the available BFRCGC studies suggest that the ITZ between CGA and the cementitious matrix is not improved by fiber addition in a uniform or automatic manner, but through a dosage and dispersion process. In Zhu et al. [22], SEM evidence showed that hydration products were attached to the BF surface, indicating that BF could participate in forming a denser local bonding zone and thereby improve the integrity of the gangue–matrix interface. In Qiu et al. [41], NMR and SEM analyses further indicated that the beneficial effect of BF was accompanied by pore refinement and a reduction in harmful porosity, suggesting that ITZ densification and matrix compactness evolved together rather than independently. Li et al. [40] emphasize that in a relatively low-strength or more porous matrix, poor BF dispersion limited the reinforcing potential of the interface, even though C-A-S-H and N-A-S-H gels could still be identified around the fibers.

These findings confirm that the macro-scale enhancement of BFRCGC is closely tied to microstructural optimization. Fiber distribution, interfacial bonding quality, and pore structure refinement directly influence compressive, tensile, and flexural performance. Practical application thus requires careful control of fiber dosage, length, and interaction with other admixtures to maximize the material’s performance under various engineering conditions.

#### 4.2.3. Concluding Remarks

Based on the reviewed studies, BF appears to contribute more consistently to tensile and flexural performance than to compressive strength in FRCGC. Based on the reported experimental evidence and the concluding interpretations of the reviewed studies, relatively favorable results were most commonly observed at BF dosages of about 0.1–0.2%, while the interval of 0.12–0.15% was more frequently associated with improved overall performance, particularly at moderate CG replacement levels. The comparatively low dosage range identified in the literature may be associated with the fine diameter, high specific surface area, and dispersion sensitivity of BF. When the dosage becomes excessive, however, the reinforcing effect tends to become less stable, likely due to fiber agglomeration, reduced workability, and the formation of internal defects.

#### 4.2.4. Knowledge Gap

Although the available studies generally support the beneficial role of BF in improving the tensile-related performance of CG concrete, several aspects still require further clarification. First, the reported optimum dosage range (0.12–0.15%) of BF is relatively narrow, and the underlying reasons may be related to fiber dispersion, interfacial characteristics, and crack-control efficiency, but the current evidence is still insufficient for a definitive interpretation. Second, most existing studies focus on short-term mechanical behavior, whereas the long-term performance of BFRCGC under durability-related exposure or repeated loading has not yet been fully established. In addition, differences in gangue source, specimen preparation, and testing procedures may contribute to the variability of reported results, which limits direct comparison across studies. Therefore, further work is needed to improve the consistency of experimental reporting and to better understand the reinforcing role of BF in different CG concrete systems.

### 4.3. Steel Fiber Reinforcement in CGC

Steel fiber (SF) reinforcement has been extensively applied across a wide range of cementitious composites owing to its superior crack resistance, load redistribution capability, and durability enhancement [71]. Compared to more recent developments such as BF or synthetic fibers, SF remains a well-established and industry-proven solution, particularly valued for its high tensile strength, stiffness, and mechanical anchorage [72,73]. In these systems, SF has been shown to significantly improve post-cracking ductility, energy absorption, and fatigue resistance, making it especially suitable for structural applications in high-rise buildings and long-span bridges [74].

While the role of SF in conventional and advanced concrete systems has been thoroughly investigated, its specific application to CGC remains insufficiently synthesized. In recent years, the use of SFRCGC has drawn attention as a means to compensate for the inherent drawbacks of CGA, such as their poor strength and weak interface bonding [75,76]. However, significant variations in the mix design, replacement ratios for coarse aggregates, fiber types, and testing methodologies used in research mean that results regarding mechanical enhancement, optimal fiber dosage, and dominant reinforcement mechanisms remain fragmented.

To provide a systematic summary of current knowledge, this review collects and compares data from nine representative studies on SFRCGC under varying gangue replacement ratios for coarse aggregates (0–100%) and SF content ratios (0–2%). Their key findings and experimental parameters are summarized in Table 7, with the aim of clarifying the strengthening patterns, parameter sensitivities, and interaction mechanisms between SF and gangue-rich matrices. This review seeks to establish a broad understanding of the reinforcement efficiency of SF in SFRCGC systems for reference when optimizing the use of sustainable materials.

#### 4.3.1. The Mechanical Properties of SFRCGC

The mechanical enhancement of SFRCGC has been widely validated across existing studies, particularly in improving crack resistance, ductility, and delaying brittle failure. However, the reinforcing effect of SF is not inherently linear or universally stable; instead, it is significantly influenced by multiple interacting factors, most notably, the CG replacement ratio for coarse aggregates and the SF dosage. The former governs the baseline strength and defect sensitivity of the concrete matrix, while the latter determines the fiber distribution density, interfacial bonding quality, and load transfer efficiency.

Previous studies have shown that SF can substantially improve CGC performance within specific replacement and dosage ranges. However, when either parameter exceeds its optimal threshold, the incremental gains often plateau or may even reverse due to issues such as poor dispersion, weak interfaces, or increased heterogeneity. Therefore, understanding the coupling effects between gangue content and fiber dosage is critical for establishing the optimal design and engineering application of SFRCGC.

To clarify these interactions, this review presents a two-dimensional analytical framework:

(1) The compensation mechanisms of SF at varying gangue replacement levels.
(2) The regulating effects of fiber dosage on mechanical performance.

All supporting data are compiled from the nine peer-reviewed studies previously mentioned, the aggregated results of which are summarized in Table 7, including key variables, test parameters, and performance trends.

#### 4.3.2. The Effects of Varying the Gangue Replacement Ratio for Coarse Aggregates

Focusing on the CG replacement ratio for coarse aggregates as the primary variable, this section evaluates how the incorporation of SF influences the compressive, splitting tensile, and flexural strength of concrete across different levels of gangue substitution (*R_cg_* (%)), as illustrated in Table 7.

In the low replacement range (0–35%), the addition of SF delivers the most noticeable improvements, especially in tensile-related properties. For instance, Ge and Cai [81] reported 0.8% SF added to a 25% aggregate replacement mix improved compressive strength from 38.3 MPa to 42.3 MPa. Karimipour [83] described that at 25% aggregate replacement, the flexural strength nearly doubled—from 3.9 MPa to 7.42 MPa—at a 1.5% fiber dosage, highlighting the toughening efficiency under low replacement conditions.

As the replacement level approaches a moderate range (35–70%), matrix strength tends to decrease due to aggregate weakening, yet SF provides substantial compensation. In Karimipour [83], the 50% replacement group with 1.5% fiber content achieved compressive and flexural strengths of 48.53 MPa and 6.86 MPa. Remarkably, Shan et al. [78] reached 106.43 MPa in compressive strength at 50% replacement by optimizing the water-to-binder ratio and admixture usage alongside 1% SF, demonstrating the potential of fiber synergy in a high-performance mix design.

In contrast, the high replacement range (≥70%) exhibited diminishing returns in strength gains, with the reinforcing effect of SF becoming marginal or even negative. In Ge and Cai [81] and Cheng et al. [42], at 100% aggregate replacement, increasing fiber content to 1.2% only led to a 1–2 MPa improvement in compressive strength (less than 5%). Qiu et al. [82] and Luo et al. [56] reported similar saturation effects, with 2% fiber addition even resulting in performance decline—e.g., Qiu, et al. [82] found that compressive strength fell from 30.04 MPa to 28.44 MPa—possibly due to fiber agglomeration, interfacial defects, and increased water entrapment.

In summary, the reinforcing efficiency of SF in FRCGC shows a clear coupling relationship with gangue replacement level and fiber dosage. Based on the representative studies summarized in Table 7, CG replacement levels for coarse aggregates were mainly distributed from 0% to 100%, while the mechanically beneficial SF dosage was most commonly reported within approximately 0.8–1.5%. Within low-to-moderate replacement ranges, SF generally produced clearer improvements in compressive and tensile-related properties, owing to more effective crack bridging and interfacial toughening. At higher replacement ratios, however, the reinforcing effect tended to become less stable, as the deterioration in gangue aggregate quality and fiber dispersion increasingly constrained strength development. Moreover, the available results indicate that the enhancement in splitting tensile and flexural behavior is often more pronounced than that in compressive strength. Karimipour [83] further suggested that SF can provide effective strength recovery and toughening under both CGA and CGS replacement conditions, although the magnitude of improvement depends on the type and level of replacement.

#### 4.3.3. The Effect of Varying Fiber Content of SFRCGC

Building upon the previous analysis of the effect of gangue replacement ratio for coarse aggregates, this section shifts focus to the role of SF dosage. Being a critical parameter in mechanical enhancement, fiber content strongly governs crack control, interface behaviors, and the overall reinforcement efficacy of FRCGC. A review of representative studies within the 0–2.0% content range is presented, highlighting key results in Table 7.

At the low content range of 0.25–0.5%, SF already demonstrates noticeable enhancement effects, particularly in splitting tensile strength. For example, in Cai et al. [20], with a 20% gangue replacement level, adding 0.5% SF raised the compressive strength from 49.95 MPa to 51.38 MPa. Additionally, in Li et al. [77], in which 0–30% aggregates replacement for coarse aggregates and 0.25–0.5% fiber dosage was examined, compressive strength values above 59–65 MPa were consistently achieved, indicating robust structural compensation. These findings suggest that even at relatively low dosages, SF can greatly improve interfacial integrity and crack propagation resistance.

In the moderate dosage range of 0.5–1.0%, the performance gains approach peak values, so this dosage range is often recommended as optimal. Cai et al. [79] demonstrated that under a full (100%) coarse gangue replacement condition, adding just 1.0% SF increased compressive strength from 35.87 MPa to 46.56 MPa and splitting tensile strength from 2.44 MPa to 3.48 MPa, exhibiting an excellent cost–performance balance. Moreover, data from Karimipour [83] revealed that in both coarse and fine aggregate replacement scenarios, SF dosages of 1.0% to 2.0% significantly improved flexural strength, with some gains exceeding 60%, suggesting strong adaptability under composite failure conditions.

When the SF dosage is increased to 1.5% and above, the improvement trend becomes less stable. In Li et al. [77], for example, the compressive strength peaked at 106.43 MPa with 1.0% fiber, but declined to 56.54 MPa with a 2.0% dosage. Cai et al. [20] similarly observed a drop in compressive strength from 42.9 MPa to 39.8 MPa when the dosage was increased from 1.0% to 1.2%. A comparable pattern was reported in Qiu et al. [82], where compressive strength fell from 30.04 MPa to 28.44 MPa as the fiber content rose from 1.0% to 2.0%, indicating a “saturation effect.” These reductions are likely attributable to fiber agglomeration, reduced workability, weaker interfacial bonding, and increased porosity, all of which contribute to local defects and diminished overall performance.

In conclusion, the reviewed studies suggest that the mechanically beneficial SF dosage in CGC is generally concentrated at 0.8–1.5% within an overall investigated range of 0–2.0%. Across the representative studies, peak compressive, splitting tensile, and flexural strengths reached 24.12–108.23 MPa, 2.67–6.20 MPa, and 6.72–25.03 MPa, respectively. Compared with compressive strength, tensile- and flexural-related properties usually exhibited more pronounced enhancement, indicating that SF is particularly effective in crack bridging and toughness improvement. When the dosage exceeded about 1.5%, however, the reinforcing effect often became less stable because of poorer dispersion, reduced workability, and increased matrix defects. Therefore, 0.8–1.5% can be regarded as the most suitable dosage range for most SFRCGC systems.

#### 4.3.4. Coupled Effects of Matrix Design and SF Reinforcement in SFRCGC

SF reinforcement in CGC is better understood as a matrix–fiber coupled response than as an isolated dosage effect. Across the representative studies, SF contents were generally investigated within 0–2.0%, but the mechanically favorable range was not fixed in an absolute sense; instead, it shifted with matrix design, aggregate quality, and service requirements. A representative example is provided by Shan et al. [78] who examined the combined effects of water-to-binder ratio, CGA content, SF content, and water-reducer dosage through an orthogonal design. Their results showed that the influence on compressive strength could be ranked approximately as water-to-binder ratio > CGA content > SF content > water-reducer dosage, and that under optimized matrix conditions, SFRCGC could achieve compressive strengths of 60–90 MPa together with a slump of 140–220 mm. This confirms that the matrix condition largely determines whether the crack-bridging role of SF can be effectively translated into strength enhancement.

This interaction is also evident in high-replacement systems. In the study of Cai et al. [20] the water–cement ratio and plasticizer dosage were kept constant (w/c = 0.40, plasticizer 5.04 kg/m^3^), allowing the influence of SF to be interpreted under a controlled matrix. Under 50% CGA replacement, the axial compressive strength of SFRCGC reached 46.62 MPa, very close to the 47.11 MPa measured for SF reinforced normal concrete. More importantly, under 100% CGA replacement, increasing SF content from 0% to 1.0–1.5% raised axial compressive strength from 35.87 MPa to 46.56–46.83 MPa, while splitting tensile strength increased from 2.44 MPa to 3.48–3.61 MPa. These results indicate that even when CGA quality imposes a clear strength penalty, a sufficiently controlled cementitious matrix still allows SF to recover much of the lost mechanical performance.

A further perspective is provided by Cheng et al. [42] whose mix design remained essentially fixed at 195 kg/m^3^ water, 390 kg/m^3^ cement, 800 kg/m^3^ sand, and 7.8 kg/m^3^ water reducer, while CGA replacement and SF dosage were varied. Their results showed that at a 25% replacement level, 0.8% SF reduced the mass loss rate after freeze–thaw cycles by 36.54% and increased the relative dynamic elastic modulus by 8.43%, whereas excessive fiber addition became less effective because of agglomeration and internal defects. This suggests that the mechanically or durably optimal fiber content is not universal, but depends on how the fiber dosage interacts with matrix compactness, CGA replacement level, and damage tolerance.

#### 4.3.5. Microstructural Characteristics of SFRCGC

**Figure 7:**
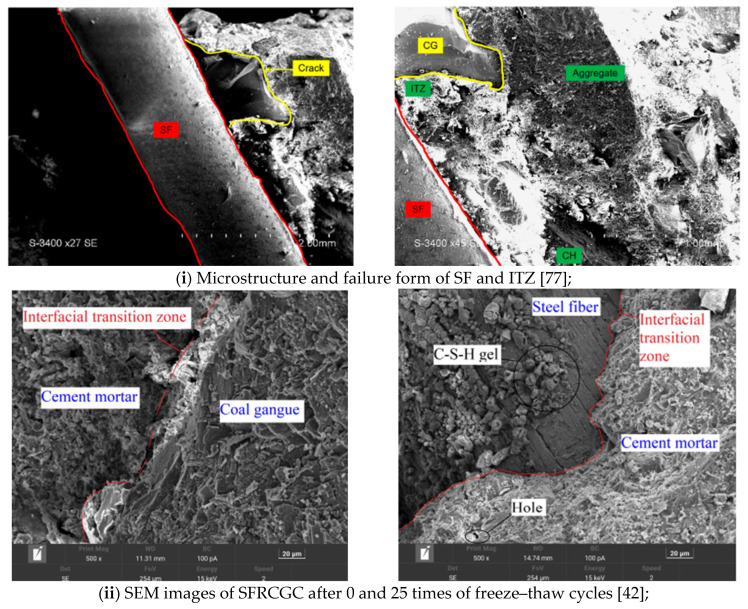

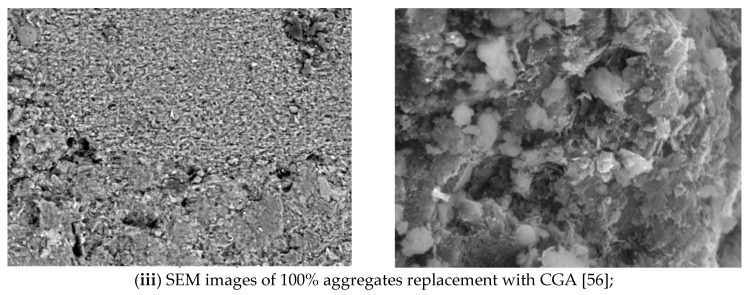
Microstructure analysis of SFRCGC.

Compared to BF, SF is a stiffer and mechanically stronger reinforcement material, offering significantly higher tensile strength and linear rigidity. While BF is known for its low density, moderate elastic modulus, and excellent chemical stability, its lower intrinsic stiffness often limits its reinforcing effectiveness under high-stress or defect-sensitive conditions. In contrast, the superior mechanical properties of SF make it particularly effective in enhancing crack resistance, flexural strength, and tensile capacity in concrete. Therefore, it is of both theoretical and practical significance to investigate the microstructural enhancement mechanisms of SF in FRCGC, especially in comparison with those of BF. To further elucidate the micro-level reinforcement mechanisms of SF in FRCGC, this section synthesizes insights from the existing literature, with particular reference to the shared observations and key differences reported in microstructural behaviors. These findings, summarized in Table 8 and Figure 7, aim to provide detailed and practical references for optimizing FRCGC in engineering applications.

Compared to BF, SF exhibits superior rigidity and mechanical strength, making it particularly effective in resisting high stress and internal material defects [84]. As summarized in Table 8, SEM analyses across multiple studies illustrate the key reinforcement mechanisms of SFRCGC. For instance, Figure 7i clearly shows that SF bridges crack within the matrix and significantly densify the interfacial transition zone. A substantial accumulation of C–S–H gel on the fiber surfaces enhances the bonding between the fibers and the cementitious matrix at the microstructural level.

From a mechanical perspective, Shan et al. [78] indicates that increasing fiber content from 0.4% to 1.2% leads to a notable rise in compressive strength, to as much as 39.82 MPa, with axial compressive strength maintained above 30 MPa. These improvements are consistent with the SEM evidence showing more effective crack bridging and ITZ compaction at optimal dosages. However, as shown in Figure 7iii, when the fiber content exceeds 1.2%, excessive clustering and void formation can occur, undermining the composite’s structural integrity and reducing mechanical performance. Shan et al. [78] examines high gangue replacement conditions (70–100%). With optimized water-to-binder ratios and superplasticizers, the inclusion of 1% SF resulted in compressive strength as high as 108.23 MPa. Karimipour [83] confirms that a dense hydration layer and effective fiber–matrix bonding suppress crack development and account for substantial strength gains, even under challenging mix conditions. Similarly, Figure 7ii,iii demonstrate that SF is particularly effective at moderate gangue replacement levels (25–50%). At fiber dosages between 0.8% and 1%, both compressive and splitting tensile strengths improved markedly, supported by SEM observations of well-bridged cracks and compact ITZs. In contrast, higher dosages (e.g., 2%) led to fiber entanglement and localized defects, limiting further performance enhancement.

In SFRCGC, the interfacial transition zone should be interpreted more specifically as the combined response of the steel fiber–matrix interface and the gangue-adjacent weak region. Available studies suggest that SF generally develops a relatively strong mechanical bond with the surrounding mortar. For example, Shan et al. [78] observed micro pits and adhered mortar debris on the pulled-out steel fiber surface, indicating that the interfacial bond was sufficiently strong to transfer stress and consume energy during pull-out. However, the weak link in SFRCGC is not always the steel fiber–paste interface itself. In Shan et al. [78], fracture in SFRCGC was found to occur mainly in the gangue concrete phase rather than at the mortar–aggregate interface, suggesting that the low stiffness and weakness of gangue could dominate failure once the steel fiber–matrix bond had been improved.

In summary, the macro-scale performance improvements observed in SFRCGC are closely linked to its refined microstructure. Crack bridging and ITZ densification emerge as the primary mechanisms of reinforcement. By appropriately regulating fiber content and the gangue replacement ratio for coarse aggregates, the full toughening potential of SF can be realized, resulting in SFRCGC materials with enhanced strength, durability, and crack resistance.

#### 4.3.6. Conclusion Remark

The reviewed studies suggest that the reinforcing effect of SF in FRCGC depends on both fiber dosage and CG replacement level. Relatively favorable performance was more commonly reported at moderate gangue replacement levels (about 35–70%) with SF dosages of around 0.8–1.0%. Within this range, SF was generally associated with improvement in compressive strength and more pronounced enhancement in tensile and flexural behavior, contributing to crack control and toughness. Microstructural evidence further suggests that these benefits are related to crack bridging and improved interfacial compactness. At excessively high gangue replacement levels, however, the reinforcing effect tended to become less stable, likely because the higher porosity and defect content of the matrix reduced fiber dispersion efficiency and stress transfer.

#### 4.3.7. Knowledge Gap

Although SF has shown the strongest overall reinforcing effect among the reviewed fibers, its governing mechanism in CG concrete still requires further clarification. In particular, it remains unclear to what extent the strengthening effect depends on fiber pull-out resistance, crack-bridging efficiency, or the quality of the CG interfacial transition zone. This issue becomes more important at high gangue replacement levels, where matrix defects may substantially reduce the effectiveness of SF reinforcement. Therefore, further studies are still needed to clarify the interaction between SF and the weak CG matrix, and to establish more reliable dosage ranges under different replacement conditions.

### 4.4. Polypropylene Fiber Reinforcement in CGC

Polypropylene fiber (PPF), a typical synthetic fiber, has attracted considerable attention in recent years for its lightness, low cost, excellent corrosion resistance, and superior dispersibility [85]. In the study of FRCGC, where the use of inferior-quality aggregates tends to increase brittleness and reduce structural reliability, PPF has come to be regarded as an effective toughening agent.

Current research predominantly explores PPF dosages in the range of 0% to 2%, under varying CG replacement levels and aggregate substitution strategies (i.e., coarse, fine, or dual replacement). Although PPF exhibits lower tensile strength and elastic modulus than inorganic fibers such as SF, its excellent distribution capability and fiber network formation confer unique advantages at the microscale level [86]. Notably, PPF has demonstrated favorable performance in early-stage crack control and fatigue resistance, particularly with low to moderate fiber content [87], where a balance between mechanical improvement and workability can be struck [88].

To systematically investigate the strengthening mechanisms of PPF in PPFRCGC, this study reviews four representative publications, covering various fiber dosages and aggregate replacement modes. Mechanical properties such as compressive strength, flexural strength, and splitting tensile strength are summarized and comparatively analyzed. Relevant key experimental data are presented in Table 9.

#### 4.4.1. The Effect of PPFRCGC with Different CG Replacement Ratios for Coarse Aggregates

According to the reviewed literature in Table 9, PPF demonstrates a notable reinforcing effect even at high replacement rates, with particularly evident benefits under full replacement conditions. In Zhu et al. [57], 100% of the coarse aggregate was replaced with CG. The control group (without fiber) exhibited an average compressive strength of approximately 43 MPa and a flexural strength of about 9.8 MPa. Upon incorporating 0.6–1.5% PPF, the compressive strength increased to a maximum of 49.7 MPa, and the flexural strength rose to 10.49 MPa—representing an improvement of 13–15%. Notably, the 0.6% dosage yielded the most balanced performance, with stable strength gains, indicating that PPF effectively enhances ductility and crack resistance under full CG replacement.

Under moderate CG replacement levels—such as the UCCA replacement range of 10–30% in Karimipour [83]—PPF exhibited consistent improvement in mechanical properties. With the incorporation of 9 kg/m^3^ PPF (approximately 1% by volume), the compressive strength declined from 38.70 MPa to 29.1 MPa as the CG content increased. However, this reduction was significantly mitigated compared to the plain concrete group. Meanwhile, the splitting tensile strength remained within the range of 3.3–4.4 MPa, and flexural strength consistently exceeded 4.5 MPa. These results highlight PPF’s capacity to maintain mechanical integrity by bridging cracks and distributing stress more effectively, even when aggregate quality is compromised.

In Wu et al. [89], concrete mixes with replacement rates of 20%, 40%, and 60% were reinforced with 0–0.3% PPF. Despite relatively low fiber content (0.1–0.3%), the compressive strength of the reinforced material increased from 25.1 MPa to approximately 29.2 MPa, with the maximum improvement nearing 16%. Even at a 60% replacement rate, 0.2% PPF maintained compressive strength at around 29 MPa, indicating that PPF can compensate for strength losses within a practical dosage range.

In summary, the reviewed studies suggest that PPF provides effective reinforcement in CGC, especially in crack resistance and flexural performance. Across the representative studies, compressive, splitting tensile, and flexural strengths reached 24.12–50.52 MPa, 1.95–5.81 MPa, and 3.85–10.49 MPa, respectively. Compared with compressive strength, tensile- and flexural-related properties generally showed more pronounced enhancement, reflecting the roles of PPF in crack arrest, ductility improvement, and interfacial toughening. The mechanically beneficial dosage was most commonly reported at approximately 0.6–0.9%, or 9–18 kg/m^3^. However, the reinforcing effect tended to be more limited at high CG replacement levels. Therefore, moderate PPF incorporation appears to provide the most suitable balance between mechanical enhancement and crack resistance in most PPFRCGC systems.

#### 4.4.2. The Effect of Varying Fiber Content Within PPFRCGC

Literature data reveal that within the 0–2% dosage range, PPF tends to exhibit a performance trend of initial improvement followed by stabilization or slight decline, particularly with regard to flexural and tensile strength enhancements.

At a moderate dosage of 0.6–1.0%, PPFCGC achieves its optimal mechanical performance. Zhu et al. [57] report that, under 100% CG replacement, the compressive strength of the control group without fiber remained between 42 and 44 MPa, while flexural strength was approximately 9.5–10.1 MPa. With 0.6% PPF addition, compressive strength increased to 49.7 MPa, and flexural strength reached 10.49 MPa—representing gains of approximately 15% and 10%, respectively. At 0.9% dosage, although compressive strength slightly fluctuated (46–48 MPa), flexural strength remained high (9.8–10.3 MPa), indicating sustained control over plastic cracking.

In contrast, a higher dosage range (1.2–1.5%) yielded diminishing returns. According to Zhu et al. [57], compressive strength in the 1.5% group declined slightly to 44–47 MPa—lower than that at 0.6% or 0.9%—and flexural strength, while still stable at 9.5–10.3 MPa, showed saturation in improvement. A similar trend was observed in Karimipour [83]: at a 30% CG replacement rate, increasing the PPF dosage from 1% to 2% yielded only a marginal compressive strength gain (from 29.1 MPa to 30.1 MPa), and splitting tensile strength increased from 3.3 MPa to 3.7 MPa. These gains were far less significant than those observed when increasing the dosage from 0% to 1%, which yielded nearly a 1 MPa improvement.

At lower dosages (<0.5%), although improvement was more limited, measurable performance enhancement was still evident. Wu et al. [89] demonstrated that increasing PPF from 0.1% to 0.3% raised compressive strength from 25.1 MPa to 29.2 MPa, especially under moderate CG replacement levels (40–60%). The strengthening effect at this stage is primarily attributed to the fiber’s ability to control early-age microcracking, making it suitable for lightweight structures or applications with high crack-resistance requirements.

Building on the analysis of PPFRCGC, some studies further explored its hybrid use with SF [90]. Compared to single-fiber systems, hybrid fiber reinforcement demonstrates more balanced improvement by combining early-stage crack resistance with enhanced load-bearing capacity. Experimental results indicate that when SF content ranges from 0.5% to 0.75% and PP fiber content from 0.2% to 0.3%, the compressive and splitting tensile strengths of concrete are generally superior to those of single-fiber groups. Specifically, compressive strength remains above 35 MPa, and splitting tensile strength consistently exceeds 2.4 MPa—a marked improvement over the plain concrete baseline. The synergy of SF and PPF causes a multiscale toughening effect in CG concrete. PP fibers help control early microcracks, while SF enhances post-cracking ductility and load-bearing capacity. This hybrid mechanism improves both crack resistance and interface integrity, providing more stable mechanical performance under varying gangue replacement rates.

#### 4.4.3. Effect of Gangue Replacement and Fiber Reinforcement on the Modulus of Elasticity

The modulus of elasticity of gangue concrete has received much less attention than strength-related properties, but the available evidence indicates that it is highly sensitive to both gangue replacement level and fiber type. Based on the results reported by Karimipour [83], replacing natural aggregates with untreated coal waste generally altered the elastic modulus in a non-monotonic manner: at low replacement levels, the modulus could be maintained or even improved, whereas further increase in gangue content led to a clear reduction because of the lower stiffness and higher porosity of coal waste aggregates.

For CGA, the elastic modulus first increased and then decreased with increasing gangue content. The maximum elastic modulus of FRCGC mixtures was obtained at around 5–10% replacement. In particular, using 1% PPF and 1% SF with 5% CGA increased the modulus by about 50% and 60%, respectively, whereas with 10% CGA, the use of 2% PPF and 2% SF increased the modulus by about 93% and 130%, respectively. A similar trend was observed for CGS. The elastic modulus reached its maximum at about 5% CGS, and fiber incorporation substantially improved stiffness recovery. When 5% CGS was combined with 2% PPF and 2% SF, the modulus increased by about 107% and 121%, respectively. However, as the replacement level continued to increase, the modulus declined, indicating that the beneficial role of fibers could not fully compensate for the weaker and more deformable gangue aggregate system.

The results of Karimipour [83] suggest that the CGA or CGS ratio is a governing factor for the modulus of elasticity, while fiber reinforcement can partly compensate for stiffness loss within a limited replacement range. Compared with PPF, SF showed a consistently stronger improvement in elastic modulus, which is consistent with its higher stiffness and stronger load-transfer capacity. Therefore, although moderate gangue replacement combined with fiber addition may preserve or even enhance stiffness, excessive gangue incorporation tends to reduce the elastic modulus of concrete.

#### 4.4.4. Concluding Remark

Compared with SF and BF, the available evidence on PPF-reinforced CG concrete is still relatively limited, and the reported results are less systematic. Existing studies generally suggest that PPF is more beneficial for crack control and flexural-related performance than for compressive strength enhancement, indicating that its main contribution is likely associated with deformation compatibility and crack development restraint rather than direct improvement of matrix load-bearing capacity. In hybrid systems, PPF may also play a complementary role by controlling fine cracks, but the overall reinforcing effect still depends strongly on the interaction with other fibers and the CG replacement level.

#### 4.4.5. Knowledge Gap

A key limitation of the current evidence is the small number of available studies on PPF in CG concrete, which makes it difficult to draw robust conclusions. In addition, the reinforcing mechanism of PPF has not yet been clearly established. It remains uncertain whether the reported improvements are governed mainly by microcrack restraint, crack-width control, toughness enhancement, or synergistic interaction in hybrid systems. Because the existing dataset is still sparse and the reported observations are not sufficiently consistent, further studies are needed before reliable performance trends and dosage recommendations can be established for PPF-reinforced CG concrete.

## 5. Durability Properties of FRCGC

CG has garnered increasing attention as a promising recycled aggregate, and its application in concrete has become a focal point in recent research. In particular, coal CGC has come into use in underground engineering scenarios such as mine roadways, shafts, and backfilling, due to its cost-effectiveness and resource utilization potential. However, CGC often exhibits inherent durability limitations arising from its high porosity and weak ITZ, which make it particularly susceptible to performance degradation under aggressive conditions such as freeze–thaw cycles, sulfate attack, and chloride ion penetration [14,91]. Studies have shown that, compared to conventional natural aggregate concrete, CGC tends to exhibit higher permeability and more rapid deterioration, limiting its applicability in cold or saline environments [12].

To address these challenges, the incorporation of reinforcing fibers such as SF and BF has been explored, aiming to improve the microstructural integrity of the concrete, reduce porosity, and inhibit crack propagation. To systematically summarize the current progress on the durability performance of FRCGC, this study selects seven representative publications covering a range of environmental conditions—including freeze–thaw, sulfate, thermal, and chloride exposure—and extracts their findings and mechanistic insights. These are detailed in Table 10.

### 5.1. Freeze and Thaw Cycling

Under freeze and thaw cycling, CGC is highly susceptible to moisture ingress and crack propagation due to its high porosity and weak ITZ. Cheng et al. [42] reported that, with a 25% gangue replacement rate and 0.8% SF content, the mass loss of the concrete after 100 freeze–thaw cycles was only 1.98%, representing a 36.54% reduction compared to the control. Additionally, the elastic modulus increased by 8.43%. This study also successfully established a Weibull distribution model to predict degradation under cyclic freezing. Qiu et al. [82] further examined the coupling effect of freeze–thaw and capillary absorption. Their results show that while the 1.0% SF group exhibited the lowest water absorption rate, water uptake still increased by a factor of 3.28 after 30 cycles, highlighting that microcrack development at the fiber–matrix interface is a key factor in freeze and thaw damage. This underscores the importance of fiber dispersion and optimal dosage to avoid the formation of preferential water pathways. Moreover, Qiu et al. [41] demonstrated that combining 0.15% BF with a binary mineral admixture system (fly ash/silica fume, F/S = 1) effectively resisted freeze and thaw damage over 300 cycles, with no visible surface deterioration. Pore structure analysis revealed a 16.89% reduction in harmful pores and a 9.19% increase in harmless pores. These findings confirm the synergistic role of BF and pozzolanic materials in improving pore refinement and mitigating freeze-induced stress cracking, making this approach especially promising for cold-region applications where long-term durability is critical.

The integration of SF and BF significantly enhances the freeze and thaw durability of FRCGC. SF improves mechanical resilience by bridging cracks and reducing mass loss, while BF optimizes pore structure and suppresses microcrack development under cyclic freezing, especially when used with mineral admixtures. However, the benefits are highly dosage-dependent—excessive fiber content may induce interface defects and facilitate moisture ingress. Therefore, a careful balance between fiber type, content, and supplementary materials is essential to ensure long-term durability, particularly in cold-region infrastructure.

### 5.2. Sulfate Attack

In underground mining environments—where sulfate exposure is prevalent and CGC is widely applied—CGC exhibits notable vulnerability. The presence of sulfates induces chemical expansion, crystal formation, and microcrack evolution, all of which compromise the material’s long-term durability. The ingress of SO_4_^2−^ reacts with hydration products such as Ca(OH)_2_ and C–S–H gel, generating expansive phases like gypsum and ettringite. These products grow within the pore structure, exerting internal pressure that accelerates crack development and surface spalling.

Li et al. [40] investigated this degradation process using an alkali-activated binder system incorporating fly ash and slag, combined with 35% CGA and 0.1% BF. Under 60 cycles of wet–dry exposure in 5% sodium sulfate solution, specimens exhibited only minor surface scaling and local salt crystallization. Compared to plain CGC, mass loss was significantly lower, and the compressive strength retention index was higher by 20.02%. Microscopic observations revealed that BF maintained strong bonding with the matrix, showing no chemical degradation. Corrosion products were predominantly observed on the outer surface of the fibers, without penetrating the fiber–matrix interface. Additionally, the N–A–S–H and C–A–S–H gel networks formed within the alkali-activated matrix contributed to a denser microstructure that effectively blocked SO_4_^2−^ ingress.

Similarly, He et al. [39] demonstrated enhanced sulfate resistance in specimens containing 40% CG and varying BF dosages. With 0.15% BF, the compressive strength retention after 30 wet–dry cycles improved by 6.21–8.50%. The authors attributed this enhancement to the formation of a “spatial skeleton” by BF during matrix hardening, which suppressed crack growth and enhanced interfacial toughness. Fibers acted as crack-bridging elements, interrupting the migration of corrosive ions toward the interfacial transition zones.

Overall, BF exhibits superior chemical stability and protective interfacial performance under sulfate attack compared to SF, particularly when used in alkali-activated systems. A hybrid design of 0.1–0.15% BF with an alkali-activated binder is therefore recommended for sulfate-exposed structures such as pavements, retaining walls, and permeable infrastructures.

### 5.3. Elevated Temperatures and Chloride Ingress

CGC undergoes significant structural and phase changes at elevated temperatures, including the evaporation of pore water, decomposition of cementitious gels, and thermally induced cracking due to differential expansion between aggregates and paste. These phenomena severely affect the material’s residual strength and crack propagation resistance. Ge and Cai [81] demonstrated that with a 1.5% SF and 20% CG replacement, the SFRCGC specimens exhibited a 10.7% increase in compressive strength and a remarkable 76.7% increase in splitting tensile strength even after exposure to 800 °C. Additionally, the maximum crack width was reduced by 29%. The post-crack load–deflection response showed secondary peak behaviors and a gradual descending branch, indicating enhanced post-peak toughness and crack-bridging effectiveness. These enhancements are attributed to the high melting point and thermal conductivity of SF, which allow it to dissipate localized thermal stress and delay microcrack coalescence. However, the study also reported sporadic spalling under sealed heating conditions, suggesting that uncontrolled vapor pressure build-up could trigger sudden failure, and highlighting the need for adequate venting in structural design. Regarding chloride resistance, Wang and Zhao [92] employed the ASTM C1202 rapid chloride permeability test and found that increasing the SF content from 0% to 2% reduced the charge passed from 2680 C to 1240 C—a 53.7% reduction. The authors attributed this to SF’s ability to refine the ITZ, hinder stress concentrations at crack tips, elongate chloride transport paths, and improve matrix–aggregate bonding. Additionally, a performance coefficient evaluation showed that a 1.5% SF ratio provided the optimal balance between durability, mechanical strength, and cost-efficiency, with a system performance index reaching 0.87.

In summary, SF offers substantial advantages in terms of residual strength retention at high temperatures and enhanced resistance to chloride ingress. These benefits make SF particularly suitable for application in underground structures, marine environments, and tunnel linings where both fire and corrosion resistance are critical. Nevertheless, fiber content should be kept below 2% to avoid reduced workability and dispersion issues.

### 5.4. Conclusion Remark

Overall, the durability of CGC can be significantly enhanced through the incorporation of appropriate fiber types and dosages. For freeze and thaw cycling, a combination of 0.8% SF or 0.15% BF with a blended mineral admixture system (fly ash to slag ratio F/S = 1) has demonstrated the ability to withstand over 300 cycles without visible damage, making it suitable for cold or cyclic wet–dry regions. Under sulfate exposure, BF shows superior chemical stability, with an optimal dosage of 0.1–0.15% effectively mitigating surface erosion and strength loss. In elevated temperature conditions, SF improves residual strength and crack resistance, with 1.5% being a recommended dosage; however, care must be taken in sealed environments where explosive spalling may occur due to internal vapor pressure. Regarding chloride ingress, a 1.5% SF dosage strikes a good balance between strength, permeability resistance, and cost-efficiency.

Despite these encouraging findings, current research on the durability of FRCGC remains fragmented and limited in scope. Future studies should aim to establish more systematic experimental frameworks and standardized evaluation methods to better guide material selection and engineering applications under complex service conditions.

## 6. Economic and Sustainability Evaluation of FRCGC

CG, as an abundant industrial by-product, inherently contributes to sustainability by reducing natural resource consumption and mitigating waste accumulation. However, the addition of reinforcing fibers, such as SF, PPF, and BF, while effective in performance enhancement, may simultaneously lead to elevated costs and embodied carbon emissions. If such increases outweigh the structural or service-life benefits obtained, the green potential of FRCGC may be significantly compromised. In light of the increasing demand for low-carbon and resource-efficient construction materials [93], evaluating the practical applicability of FRCGC requires consideration of factors beyond mechanical performance. Although the incorporation of fibers improves the strength, ductility, and durability of FRCGC, these gains may be offset if they negate the economic or environmental benefits of gangue. Hence, a more holistic assessment that considers both the economic and environmental viability of FRCGC is warranted. This is all the more necessary when CG is used as a large-scale substitute for natural aggregates—its benefits must be preserved, not negated by costly or carbon-intensive reinforcement strategies.

### 6.1. Economic and Environmental Indicators

To comprehensively evaluate the environmental and economic adaptability of FRCGC beyond its structural performance, this study introduces two key indicators: the Carbon Emission Index (CEI) and the Cost Coefficient Index (CCI). These metrics quantify the environmental burden and material cost per unit of mechanical strength delivered by the concrete, providing a more practical lens for examining material sustainability, expressed as:

(1) $$CEI=\;\frac{E_{\mathrm{total}\;}}{f}\left(\;kg(CO_{2})/MPa\right)$$

(2) $$CCI\;=\;\frac{C_{\mathrm{total}\;}}{f}\left(USD/MPa\right)$$

where

*f* is the mechanical performance parameter (e.g., 28-day compressive, splitting tensile, or flexural strength, in MPa); *C_total_* is the total material cost per cubic meter of concrete (USD/m^3^); *E_total_* is the total carbon emissions per cubic meter (kg CO_2_/m^3^), calculated as:

(3) $$E_{total}=\sum_{i=1}^{n}\left(m_{i}\times e_{i}\right)$$

(4) $$C_{total}=\sum_{i=1}^{n}\left(m_{i}\times c_{i}\right)$$

With:

*m_i_*: dosage of the *i-th* constituent material (kg/m^3^);

*e_i_*: emission factor of the *i-th* material (kg CO_2_/kg);

*c_i_*: price factor of the *i-th* material (USD/kg);

*n*: number of distinct materials in the concrete mix.

To illustrate the application of the CEI–CCI evaluation framework, a case study was conducted using experimental data from Karimipour [83], which systematically compared the performance of CG concrete reinforced with SF and PPF. Unlike orthogonal test designs, this dataset includes a full factorial combination of different fiber types and CG replacement ratios for coarse aggregates, offering a robust basis for performance–cost–carbon trade-off analysis. Table 11 summarizes the unit cost and emission factors of the key mix parameters.

In the present analysis framework, the embodied carbon of CG was taken as 0.007 kg CO_2_ [50]. By contrast, the material cost of CG was simplified as 0 under a mine-site or near-site utilization scenario. This treatment reflects the source-level nature of coal gangue as a waste-derived material generated during coal mining and washing. In practice, when not reutilized, coal gangue often requires the mining operator to bear the cost of removal, storage, or disposal, and therefore behaves more like a waste burden than a conventional market-priced raw material. For this reason, the source material cost of CG was not assigned a positive value in the present comparison, and 0 was used as a simplified placeholder under the specified scenario. It should be noted that this simplification does not imply that the full utilization process of CG is free of environmental or economic burdens. Additional impacts associated with crushing, screening, handling, transportation, and quality control were not explicitly included within the present system boundary. Therefore, the resulting CEI–CCI comparison should be interpreted as a scenario-based simplified comparison under mine-site utilization conditions, intended to reflect the relative changes in cost and carbon effects associated with fiber incorporation, rather than as a full life-cycle assessment or a market-based cost accounting model.

### 6.2. Case Study

**Figure 8:**
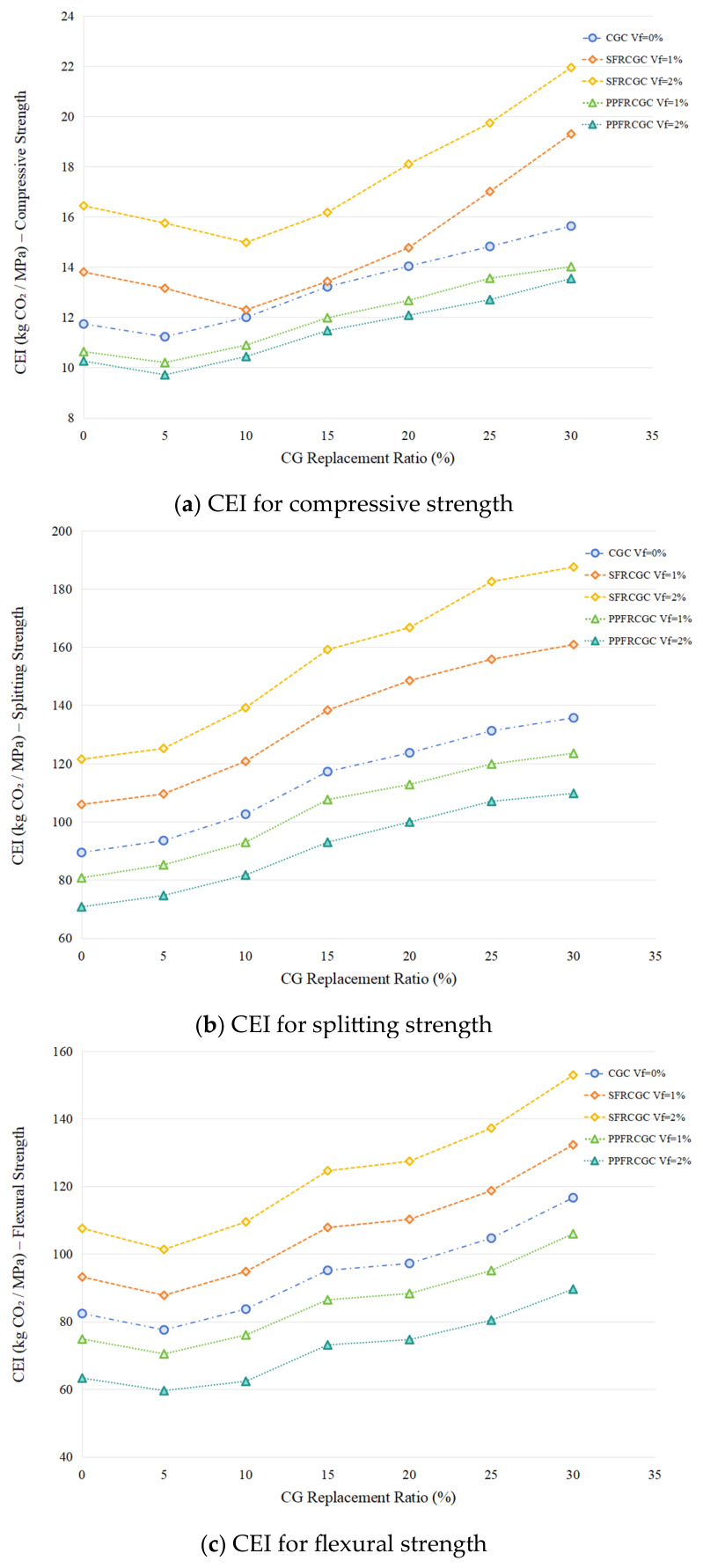

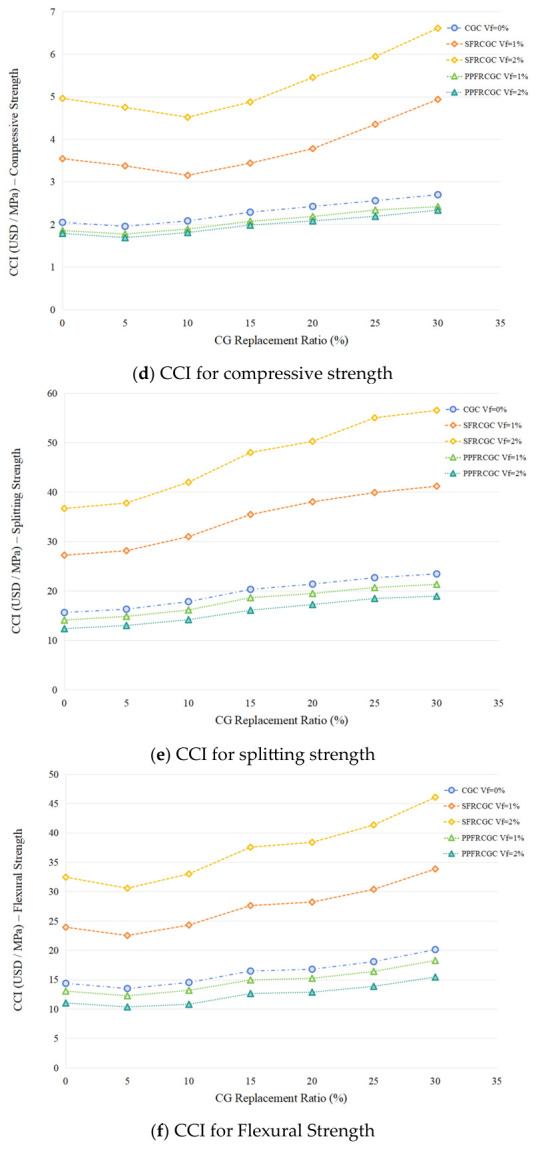
CCI and CEI for different fiber types and CG replacement ratios based on Karimipour [83].

To further evaluate the integrated performance of FRCGC, a case study was conducted based on the experimental results from Karimipour [83]. His study systematically investigated the effects of SF and PPF under varying CG replacement ratios for coarse aggregates, providing a comprehensive dataset for analysis. Using the mix proportions reported in his work, and applying the parameters in Table 11 along with Equations (1)–(3), the corresponding values of CCI and CEI were calculated. These values were used to generate Figure 8, which presents the performance–cost–carbon relationships of different CG–fiber combinations.

### 6.3. Environmental Efficiency Evaluation Based on CEI

As illustrated in Figure 8a–c, the CEI exhibits a consistent upward trend with increasing fiber content, particularly in the SFRCGC series. This is primarily attributed to the inherently high carbon emission factor of SF, which substantially elevates the total embodied carbon of the mix. As a result, both SFRCGC-1% and SFRCGC-2% configurations demonstrate significantly higher CEI values compared to the control CGC and PPFRCGC groups across all mechanical performance metrics. When holding fiber content constant, CEI also increases with higher CG replacement ratios, reflecting the diminishing marginal performance gains associated with greater use of CG. This trend suggests a decline in carbon efficiency at elevated replacement levels, highlighting the need to balance sustainability against mechanical integrity.

Among all configurations, the PPF series consistently shows the lowest CEI values across compressive, splitting tensile, and flexural strength dimensions, indicating a superior environmental–mechanical balance. Furthermore, the SFRCGC-1% series outperforms the SFRCGC-2% group in terms of CEI, underscoring that 1% SF is often sufficient to realize substantial performance gains without incurring disproportionate environmental penalties. This is especially evident in the splitting tensile strength dimension, where SFRCGC-2% yields diminishing returns relative to its carbon cost. Notably, PPF-2% exhibits the lowest CEI under the flexural strength index, probably due to its enhanced crack control and toughness. This confirms its suitability for applications requiring ductility with minimal environmental burden.

### 6.4. Economic Efficiency Evaluation Based on CCI

According to Figure 8d–f, higher fiber content leads to a noticeable increase in CCI. In particular, SFRCGC at 2% exhibits the highest values. This highlights the significant cost burden associated with SF usage, primarily due to its high unit price. In contrast, both PPFRCGC groups (1% and 2%) maintain the lowest CCI values throughout, demonstrating greater economic efficiency. Moreover, CCI tends to increase with higher CG replacement ratios. This pattern implies that while CG helps reduce raw material costs, its marginal contribution to mechanical strength decreases at higher replacement levels, ultimately raising the cost per unit strength. PPFRCGC-2% exhibits the most favorable performance-to-cost balance, particularly in flexural and splitting strength dimensions. For example, at 30% CG replacement, its flexural CCI remains below 15 USD/MPa, while SFRCGC-2% exceeds 45 USD/MPa. This suggests that the economic efficiency of PPF remains stable even under high replacement conditions. Additionally, SFRCGC-1% outperforms SFRCGC-2% in all strength categories in terms of cost effectiveness, underscoring the diminishing returns of higher SF dosages. This reinforces that moderate fiber content—especially 1%—is preferable when balancing strength improvement against material cost.

### 6.5. Concluding Remark

Overall, the CEI and CCI results show that the sustainability of FRCGC depends on achieving a balance between mechanical improvement and the additional carbon and cost introduced by fiber incorporation. The PPF series generally exhibits lower CEI and CCI values, indicating better environmental and economic efficiency, while the SF series, especially at 2% dosage, shows markedly higher carbon and cost burdens. In contrast, SFRCGC-1% performs more favorably than SFRCGC-2% in both CEI and CCI, suggesting that moderate SF dosage is more efficient than further dosage increase. Notably, PPFRCGC-2% achieves the most advantageous overall balance under the flexural index, confirming its potential for sustainable FRCGC design where toughness and crack resistance are required. However, these findings should be interpreted cautiously, as the present sustainability comparison relies primarily on a single reference dataset and simplified boundary assumptions. The results therefore provide a useful case-based illustration of performance–cost–carbon trade-offs, but their broader generalizability remains limited.

## 7. Applications of FRCGC

FRCGC is being increasingly considered for engineering use because CG is abundant near mining regions, and fibers offer a direct and practical way to offset the weak tensile behaviors and brittle cracking caused by porous and variable gangue aggregates. Current research emphasis is clearly application-driven: fibers are mainly used to improve crack control, toughness, and damage tolerance, rather than to pursue large gains in compressive strength [95,96].

At the structural scale, the member-level evidence for fiber-reinforced CG concrete is still concentrated in a few typical forms. For flexure-dominated members, studies on beams show improved ductility, energy absorption, and serviceability-related behaviors such as crack control when SF are introduced [20,79]. Under impact type actions, fibers are mainly used to reduce cracking and enhance damage controllability, which supports potential applications in slabs, pavements, and mining-related protective elements [20,97]. For compression combined with bending, tests on eccentrically loaded columns indicate that fibers contribute mainly through crack bridging and post-cracking load transfer, while the gangue replacement level still governs the overall deformation response [98,99,100]. In contrast, many structural studies on CG concrete rely on confinement or composite systems, and fiber-based member evidence beyond SF remains relatively limited.

Overall, the practical use of fiber-reinforced CG concrete is best positioned where crack control and toughness are decisive for serviceability and repairability, while moderate gangue replacement and controlled fiber dosage help maintain workability and dispersion quality. A conservative recommendation is to treat fibers as a functional upgrade strategy for structural members, and to avoid relying on fibers alone when very high gangue replacement is targeted, unless the structural form provides additional stability.

## 8. Conclusions and Recommendations

This review provides a comprehensive synthesis of the mechanical, durability, and sustainability-related performance of low-carbon FRCGC by comparing different fiber types across varying CG replacement ratios and jointly examining cost and embodied carbon, with the aim of identifying effective reinforcement strategies and practically applicable mix designs for energy-efficient and sustainable construction. The key findings and summaries from this review are as follows:

Within a CG replacement range of 25–40% (and up to 50% in some mixes), FRCGC can achieve 30–50 MPa compressive strength, with 10–70% improvements in splitting tensile and flexural strengths compared with unreinforced CGC, provided that fiber dispersion and workability are properly controlled.

Increasing fiber dosage generally enhances strength and toughness, but the incremental gains diminish beyond intermediate levels; excessive contents cause poor workability and non-uniform fiber distribution, which may offset or reverse the mechanical benefits.

Fiber systems contribute in complementary ways: SF primarily enhances load-carrying capacity and post-cracking behaviors, performing most effectively at 0.8–1.0% dosage and 25–40% gangue replacement. Polypropylene fibers provide efficient control of early-age and fine cracking, particularly in mixes with higher gangue contents, with effective dosages in the range of 0.6–1.0%. BF, used at relatively low dosages (0.12–0.15%), contributes most clearly to durability improvement and microstructural refinement, especially under freeze–thaw cycling and sulfate attack.

When evaluated using compressive-strength-based indices of cost (CCI) and embodied carbon (CEI), mixes with moderate gangue contents and fiber dosages maintain the inherent low-cost and low-carbon advantages of CGC while achieving significant improvements in mechanical performance and durability. In contrast, high fiber dosages or energy-intensive pretreatment procedures tend to erode these sustainability benefits.

### Future Research and Practical Recommendations

Future progress of fiber-reinforced CG concrete will depend on turning scattered laboratory evidence into transferable engineering guidance. First, CGA should be classified and graded using a unified set of physical indices, so that source variability can be managed through quality control and reliable mix selection. Second, durability evidence needs to move from single factor tests to coupled and realistic exposure scenarios that better represent field service, supported by longer-term monitoring of deterioration and performance retention. Third, sustainability assessment should adopt life cycle thinking by linking cost and embodied carbon efficiency to performance gains, allowing pretreatment routes, fiber systems, and gangue sources to be compared on a consistent basis. Fourth, more engineering scale validation is required through pilot and full-scale demonstrations in mining and underground infrastructure, with outcomes used to calibrate practical design parameters and construction procedures. Finally, shared datasets and model-driven tools, combining experimental databases with numerical modeling and optimization, should be developed to support region-adaptable mix design that balances mechanical performance, serviceability, cost, and carbon.

## Figures and Tables

**Table 1:** Summary of physical properties of CG reported in studies from China.

Apparent Density	Bulk Density	Water Absorption	Crushing Value	Ref.
(kg/m^3^)	(kg/m^3^)	(%)	(%)	
2653	1489	3.15	9.9	Zhou et al. [33]
2689	1201	11.4	18.1	Zhang et al. [34]
2640	-	1.8	22.6	Zhang et al. [35]
2090	-	4.92	19	Guan et al. [36]
2712	-	1.7	16.8	Ma et al. [37]
2620	1612	3.9	18.6	Duan [38]
2210	-	2	17	Zhu et al. [22]
2589	-	1.1	20	He et al. [39]
2635.26	-	2.45	10.03	Li et al. [40]
2247–2291	-	7.14–7.38	17.7–18.9	Qiu et al. [41]
2106	-	-	8.7	Cai et al. [20]
2510	-	-	17.64	Cheng et al. [42]

**Table 2:** Summary of physical properties of CG reported in international studies.

Specific Gravity	Liquid Limit	Plastic Index Values	Optimum Moisture Content (%)	Hydraulic Conductivity Values (m/s)	Country	Ref.
1.74–1.88	32–39	10–15	15–19.8	—	India	Okagbue and Ochulor [43]
2.04	27.2	9.5	10.4	10^−8^ to 10^−9^	Australia	Indraratna et al. [44]
2.04	27.2	9.5	10.4	10^−8^ to 10^−9^	Australia	Indraratna et al. [45]
2.13	27.2	9.5	10.4	10^−8^ to 10^−10^	Australia	Rujikiatkamjorn et al. [46]
2.27	27.7	10.7	9.7	10^−9^	Australia	Heitor et al. [47]
2.54	28.3	11.1	17.8	10^−9^ to 10^−10^	China	Wu et al. [48]
1.4–2.6	45.3–60	—	—	10^−5^ to 10^−8^	Australia	Shokouhi and Williams [49]
1.98–2.54	28	—	15–17	10^−5^ to 10^−6^	India Australia	Ashfaq et al. [50]

**Table 3:** Summary of chemical properties of CG.

Ref.	Country	SiO_2_	Al_2_O_3_	Fe_2_O_3_	CaO	MgO	TiO_2_	K_2_O	SO_3_	Na_2_O	Loss on Ignition
Indraratna [52]	Australia	60	20	5	0.9	1.1	0.8	1.3	/	/	/
Wu et al. [48]	China	60.38	24.73	5.76	0.81	1.37	1.36	4.31	0.11	0.89	/
Ashfaq et al. [50]	India	52.7	22.6	6.37	3.45	1.72	0.98	2.68	0.53	0.75	8.22
Okagbue and Ochulor [43]	Nigeria	71.8	14.32	3.13	0.08	0.15	2.15	0.45	/	0.08	7.37
Qureshi et al. [53]	Pakistan	31.37	20.03	3.21	0.2	0.89	1.35	0.84	0.01	0.26	41.84
Jabłońska et al. [54]	Poland	58.95	20.5	6.63	0.35	1.93	1.05	3.19	0.17	0.54	5.5
Skarżyńska [55]	Spain	52	19	6.05	2.7	1.2	1.15	3.1	0.8	0.7	13.5
Skarżyńska [55]	United Kingdom	43	21	6.5	2.1	2	0.75	3	3.85	0.7	18.5
Cai et al. [20]	China	48.76	29.51	8.02	/	2.03	0.49	1.54	/	/	/
Luo et al. [56]	China	68.32	17.42	4.43	2.31	1.25	0.84	2.5	0.31	1.5	10.79
Zhu et al. [57]	China	57.92	22.99	5.29	7.58	0.9	1.43	1.66	/	1.14	/
Mean Value		55.02	21.10	5.49	2.05	1.32	1.12	2.23	0.83	0.73	15.10
Standard Deviation		11.37	3.95	1.49	2.26	0.57	0.45	1.17	1.36	0.43	12.56
Coefficient of Variation		0.21	0.19	0.27	1.10	0.43	0.40	0.52	1.65	0.59	0.83

**Table 4:** Summary of key findings of untreated CG.

Aspect/Property	Key Conclusion	Limitation
Physical Properties	Bulk density, porosity, water absorption and crushing strength of CG from different regions (e.g., China, India, Pakistan, Nigeria) have been compiled and compared, providing a basic envelope for its use as natural aggregate replacement.	Long-term physical stability under representative service exposures (e.g., freeze–thaw, wet–dry cycles) is rarely quantified, limiting durability-related screening for structural use.
Chemical Composition	Major oxides (SiO_2_, Al_2_O_3_, CaO, Fe_2_O_3_) and loss on ignition (LOI) have been widely reported and statistically compared among regions.	Trace elements and potentially hazardous constituents are less reported, and their implications for durability, leaching and environmental safety remain insufficiently evaluated.
Regional Variability	Differences in physical and chemical properties among CG from different countries and mining areas have been identified.	No unified classification or standardized quality grading system is available, and inter-study comparisons are often confounded by inconsistent test methods and reporting.
Reactivity/Activation Potential	Basic pozzolanic reactivity of certain gangue (e.g., high SiO_2_/Al_2_O_3_ samples) has been assessed.	Activation mechanisms and reaction kinetics remain unclear for different gangue types, particularly under practical curing regimes and field-relevant conditions.
Suitability as CGA	Preliminary screening for suitability in structural or lightweight applications based on density and strength.	Systematic criteria for grading, impurity control, moisture conditioning and performance prediction are not well established, limiting design-oriented selection for structural applications.
Microstructural Characteristics	Initial SEM/EDS studies on particle morphology and microstructure.	Correlation between microstructure and macroscopic properties is not fully explored, especially for gangue from less-studied regions.

**Table 5:** Summary of mechanical properties of BFRCGC.

Ref.	*V_f_* (%)	*R_cg_* (%)	*f_c_* (MPa)	*f_t_* (MPa)	*f_fl_* (MPa)	Key Findings	Main Conclusions
Zhu et al. [22]	0.10–0.18	40%	30.16–34.52	2.02–2.41	3.28–3.71	Compressive strength increased initially and then declined, peaking at 34.52 MPa with 0.15% fiber content. Splitting and flexural strength generally increased but decreased at higher dosages.	An optimal dosage (0.15%) was identified. Overdosing may cause fiber agglomeration or weaken the ITZ. Dosage should be controlled to balance mechanical enhancement and workability.
He et al. [39]	0.05–0.25	40%	32.46–34.63	2.41–2.80	4.01–4.53	Compressive strength remained above 32 MPa; splitting and flexural strength improved significantly, with optimal results at 0.15% fiber and 18 mm length.	Fibers contributed more to tensile and flexural performance. High dosages (>0.2%) may cause dispersion issues; adjusting fiber length enhances performance.
Li et al. [40]	0.05–0.25	35%	18.23–24.12	1.81–2.95	—	Target strength was low (~21.84 MPa), and fiber-induced improvement was limited. Slightly better performance at 0.1% content and 12 mm length.	Large fiber limited dispersion and toughening effect. In low-strength matrices, the potential of BF is difficult to realize; optimization of mix design and mixing process is recommended.
Qiu et al. [41]	0.12–0.18	40%	31.42–33.88	2.37–2.44	3.69–3.79	Compressive strength fluctuated between 31 and 34 MPa. Splitting and flexural strength changed slightly with fiber content.	Fiber reinforcement was limited, possibly due to matrix composition or test parameters. Reassessment under higher-strength matrices or improved dispersion may better reflect fiber effectiveness.
Yang and Zha [64]	0.05–0.30	50%	38.77–50.02	1.83–3.44	2.47–3.79	High compressive strength observed (up to 50.02 MPa at 0.20% and 12 mm). Splitting and flexural strength did not increase synchronously. For instance, 0.30% at 12 mm reached 3.44 MPa in splitting, but only 2.79 MPa in flexural strength.	Higher fiber content increased mixing difficulty, possibly reducing performance in some cases. BF can further enhance high-strength concrete, but different fiber combinations show varied effects on tensile and flexural properties.

Note: *R_cg_* is the CG replacement level; ***V_f_*** is the fiber content; ***f_c_*** is the compressive strength; ***f_t_*** is the splitting tensile strength; and ***f_fl_*** is the flexural strength.

**Table 6:** Summary of microstructure analysis of BFRCGC.

Ref.	Key Microstructural Evidence	Focus and Differences	General Consensus
Zhu et al. [22]	-	- BF bridges crack and improve toughness - EDS shows hydration products on fiber surface enhance ITZ - Optimal dosage ≈ 0.15%, excess causes agglomeration	• BF bridges cracks and improves ITZ: confirmed by all studies. • Optimal dosage ≈ 0.12–0.15%; excess causes agglomeration and voids. • ITZ/pore refinement is key: SEM, EDS, NMR confirm reduced macro-voids and densified ITZ. • BF mitigates CGA weakness via crack control.
He et al. [39]	Figure 6i	- Quantifies effect of fiber length (6/12/18/24 mm) on frost and sulfate resistance - Best at 18 mm; shorter fibers pull out, longer fibers entangle	
Li et al. [40]	Figure 6ii,iv	- Uses alkali-activated and pervious concrete with CG - BF reduces pore connectivity and sulfate attack - SEM/EDS reveal C-A-S-H and N-A-S-H gels anchored to fibers	
Qiu et al. [41]	Figure 6iii	- NMR/SEM show refined pores and reduced harmful porosity - Strong antifreeze effect via fiber bridging and pozzolanic filling - Optimal BF ≈ 0.15%	

**Table 7:** Summary of mechanical properties of SFRCGC.

Ref.	Type of *R_cg_*	*R_cg_* (%)	*V_f_* (%)	*f_c_* (MPa)	*f_t_* (MPa)	*f_fl_* (MPa)	Key Findings	Main Conclusion
Cai et al. [20]	CGA	0%, 50%, 100%	0.0–1.5%	49.67–59.1	2.84–3.71	—	*f_c_* and *f_t_* increased with *V_f_* up to 1.5% under all replacement levels.	SF consistently enhance mechanical strength at different *R_cg_*; 1.5% *V_f_* is dosage optimal.
Li et al. [77]	CGA + CGS	CGC: 20–40%; CGS: 10–30	0.4–1.2%	32.15–39.82	—	—	Mechanical performance improved with increasing *V_f_* 0.8–1.2% most effective under dual replacement.	Positive correlation between *V_f_* and performance; effective compensation under dual replacement.
Shan et al. [78]	CGA	0%, 30%, 50%, 70%, 100%	0.0–1.0%	48.92–108.23	—	—	Nonlinear improvement in *f_c_* up to 108.23 MPa with 1% *V_f_*.	SF significantly enhance compressive strength at high replacement
Cai et al. [79]	CGA	0%, 50%, 100%	0–1.5%	35.87–47.11	2.44–3.61	—	At 100% replacement of *R_cg_*, 1.5% fiber raised *f_c_* from 35.87 MPa to 46.83 MPa.	SF effectively offset strength loss in high *R_cg_* and improve interface toughness.
Li et al. [80]	CGA + CGS	CGC: 25–45%, CGS: 15–35%	0–0.9%	29.88–35.49	2.21–2.67	—	Fiber addition improved splitting strength up to 2.67 MPa;	The enhancement in cracking resistance was more pronounced than that in *f_c_*, with the optimal *V_f_*. observed at 0.9%.
Ge and Cai [81]	CGA	0%, 20%, 40%, 60%	0–1.5%	42.39–57.54	2.60–6.01	—	Significant improvement in both *f_c_* and *f_t_* with *V_f_*. of 0.5–1.5%.	*f_t_* improvement is more prominent than *f_c_* under low-to-moderate *R_cg_*.
Cheng et al. [42]	CGA	0%, 25%, 50%, 75%, 100%	0–1.2	29.5–43.7	2.44–3.41	—	Performance enhancement observed at all replacement levels, most effective of *R_cg_* at 25–50%.	Optimal effect observed at moderate gangue levels and 0.8% fiber content.
Qiu et al. [82]	CGA	22.7–42.3%	0–1.5	38.8–39.9	—	—	*f_c_* increased slightly with fiber and replacement; impact was limited.	Limited strength improvement; mix dominated by aggregate combination.
Luo et al. [56]	CGA	0%, 25%, 50%, 75%, 100%	0–2.0	25.56–86.78	—	5.11–25.03	Significant improvements in *f_c_* and *f_fl_*, peaking at *V_f_*. of 1%.	Up to 70% improvement in strength at high replacement; 1% optimal, 2% showed saturation.
Karimipour [83]	CGA	0%, 5%, 10%, 15%, 20%, 25%	0–2%	27.54–50.14	3.11–6.20	3.90–7.42	Marked strength gains in *f_c_* and *f_fl_* at 1.5% fiber content.	1.5% fiber yielded optimal performance under all type of *R_cg_*.
Karimipour [83]	CGS	0%, 5%, 10%, 15%, 20%, 25%	0–2%	24.12–50.52	2.41–5.73	4.10–6.72	Effective toughness and crack control observed in fine aggregate replacement scenarios.	In CGS replacement scenarios, SF exhibit outstanding toughening and crack resistance performance, making them well-suited for engineering applications with elevated durability requirements.

Note: *R_cg_* is the CG replacement level; ***V_f_*** is the fiber content; ***f_c_*** is the compressive strength; ***f_t_*** is the splitting tensile strength; and ***f_fl_*** is the flexural strength.

**Table 8:** Summary of microstructure analysis of SFRCGC.

Ref.	Key Microstructural Evidence	Focus and Differences	General Consensus
Li et al. [77]	Figure 7i	-SEM/Energy Dispersive Spectroscopy(EDS) confirms C–S–H gel formation around SF at the gangue–cement interface, enhancing ITZ density and crack bridging. -Excessive fiber leads to clustering and voids. This study recommends a lower optimal fiber dosage than other literature.	All reviewed studies consistently affirm that SF play a key role in bridging cracks and reinforcing the ITZ in CG concrete. SEM images reveal that SFs are embedded across microcracks and coated with hydration products such as C–S–H gels and AFt, which enhance interfacial bonding. The pozzolanic activity of gangue contributes to interface densification when used at moderate replacement rates. Moreover, SF addition helps improve the overall compactness of the matrix, smoothen ITZ boundaries, and delay crack propagation. However, nearly all studies also report that excessive fiber content (typically >1.5%) can cause clustering, create voids, and reduce the efficiency of crack control. These observations underline the importance of optimizing fiber dosage to balance mechanical performance and structural stability.
Shan et al. [78]	-	-Post-orthogonal SEM analysis shows that higher SF content effectively suppresses microcrack growth and improves ITZ compactness, reducing local porosity. -Compared to Li et al. [77], this study supports a higher optimal fiber dosage and focuses solely on SF without considering hybrid or alkali-activated systems, as explored in Karimipour [83].	
Cheng et al. [42]	Figure 7ii	SEM results under freeze–thaw conditions reveal widened ITZ cracks, which are mitigated by SF through delayed delamination and crack propagation, reducing strength degradation.	
Luo et al. [56]	Figure 7iii	-In reactive powder concrete systems, SEM shows that high gangue content leads to increased interface voids, but SF helps mitigate microstructural damage. -Strength loss is linked to low w/b ratios and gangue-related defects.	
Karimipour [83]	-	-This study first explores hybrid fiber reinforcement (SF + PPF) in CGC. -SEM reveals that PPF enhances crack resistance while SF boosts strength, jointly contributing to synergistic toughening. -It contrasts with the single-fiber approaches of other refs, proposes a different optimal dosage	

**Table 9:** Summary of mechanical properties of PPFRCGC.

Ref.	Type of *R_cg_*	*R_cg_* (%)	*V_f_* (%)	*f_c_* (MPa)	*f_t_* (MPa)	*f_fl_* (MPa)	Key Findings	Main Conclusion
Zhu et al. [57]	CGA	100%	0–1.5%	42.8–49.7	–	9.52–10.49	When 0.6–0.9% PPF is added, both compressive and flexural strengths are significantly improved; effects become unstable beyond 1.2%.	Moderate PPF dosage (0.6–0.9%) significantly enhances flexural performance, indicating a promising toughening effect.
Wu et al. [89]	CGA	20–60%	0–0.3%	25.1–43.2	–	–	At a 60% CG replacement rate, even with 0.3% PPF, the increase in compressive strength is limited.	The reinforcing effect of PPF is relatively limited at high CG replacement levels, making it more suitable for low to moderate substitution scenarios.
Li et al. [90]	CGA and CGS	CGA + CGS ≈ 50–80%	0.1–0.4%	32.4–37.2	1.95–2.8	–	With increasing dosage, splitting tensile strength shows an overall rising trend, peaking at 2.8 MPa.	The combined use of PPF and SF can synergistically improve the crack resistance of concrete.
Karimipour [83]	CGA	0%, 5%, 10%, 15%, 20%, 25%	0–18 kg/m^3^	29.1–48.6	3.30–5.81	3.85–7.42	Adding 9 kg/m^3^ and 18 kg/m^3^ PPF improves all strength metrics, with larger gains at higher dosages.	PPF demonstrates stable toughening performance in coarse aggregate replacement systems, with peak flexural strength achieved at 18 kg/m^3^.
Karimipour [83]	CGS	0%, 5%, 10%, 15%, 20%, 25%	0–18 kg/m^3^	24.12–50.52	2.12–5.73	4.10–6.72	In fine aggregate replacement systems, enhancements in splitting and flexural strength are more pronounced.	PPF shows better crack resistance adaptability in fine aggregate replacement, making it suitable for improving ductility and interfacial toughness.

Note: *R_cg_* is the CG replacement level; ***V_f_*** is the fiber content; ***f_c_*** is the compressive strength; ***f_t_*** is the splitting tensile strength; and ***f_fl_*** is the flexural strength.

**Table 10:** Summary of durability properties of FRCGC.

Ref.	Durability Factor Studied	Material Composition	Key Findings	Key Analysis
He et al. [39]	Freeze–thaw cycles, sulfate attack	40% CG + BF (0.05–0.20%, 18 mm)	Freeze–thaw strength loss reduced by 36.76–46.90%; sulfate resistance improved by 6.21–8.50%	Fibers refined pore structure, formed spatial frameworks, and bridged cracks to mitigate freeze–thaw degradation while enhancing ITZ compactness under sulfate exposure
Ge and Cai [81]	High-temperature durability	20–60% CG + SF (0–1.5%)	At 800 °C, compressive strength increased by 10.7%, splitting tensile strength by 76.7%, and crack width reduced by 29%	SF delayed microcrack propagation at elevated temperatures and promoted early-stage hydration; risk of explosive spalling observed at 800 °C
Qiu et al. [41]	Freeze–thaw resistance	40% CG + BF (0.12–0.18%) + FA and SF	No apparent damage after 300 cycles; harmful pores decreased by 16.89% and harmless pores increased by 9.19%	Combination of mineral admixtures and fibers improved pore structure and enhanced frost resistance, suitable for cold-climate applications
Cheng et al. [42]	Freeze–thaw + damage modeling	0–100% CG + SF (0.0–1.2%)	Mass loss reduced by 36.54%; elastic modulus increased by 8.43%; deflection reached 5 mm; SEM showed crack width of 62.3 μm	Optimal performance at 0.8% SF; a Weibull model was proposed to predict freeze–thaw deterioration quantitatively
Qiu et al. [82]	Freeze–thaw cycles and water absorption	SFRCGC with fiber content (0–1.5%)	Water absorption increased by a factor of 3.28 after cycling; 1.0% SF group showed lowest absorption; strong correlation (R^2^ > 0.91) established	Fiber–matrix interfacial degradation facilitated water ingress during cycling; water absorption was a sensitive indicator of durability loss
Li et al. [40]	Sulfate attack (wet–dry cycling)	35% CG + BF (0.05–0.2%) + alkali-activated binder	Low mass loss after 60 cycles; corrosion resistance coefficient increased by 20.02%	BF improved interfacial bonding; alkali-activated matrix enhanced compactness and resistance to sulfate erosion, suitable for permeable concrete systems
Wang and Zhao [92]	Chloride ion permeability	SFRCGC with fiber content (0–2%)	Charge passed reduced from 2680 C to 1240 C (53.7% decrease); optimal dosage at 1.5%	SF significantly improved ITZ density; 1.5% content was the most effective in balancing strength, durability, and economic efficiency

**Table 11:** Material unit costs and carbon emission factors.

Material	Unit Price (USD/kg)	Carbon Emission Factor (kg CO_2_/kg)	Ref.
Cement	0.1344	0.92	[18,94]
Water	≈0	≈0	
Sand	0.0065	0.0015	
Gravel	0.0109	0.0285	
SF	1	2.2	
PPF	0.9	1.85	

## Data Availability

No new data were created or analyzed in this study. Data sharing is not applicable to this article.
